# The peristomatic structures as a source of systematic characters in the genus *Lithobius* Leach, 1814 (Myriapoda, Chilopoda)

**DOI:** 10.3897/zookeys.741.21706

**Published:** 2018-03-07

**Authors:** Anne-Sarah Ganske, Gregory D. Edgecombe, Nesrine Akkari

**Affiliations:** 1 Natural History Museum Vienna, 3rd Zoological Department, Burgring 7, 1010 Vienna, Austria; 2 The Natural History Museum, Department of Earth Sciences, Cromwell Road, London SW7 5BD, UK; 3 University of Vienna, Department of Integrative Zoology, Althanstraße 14, 1090 Vienna, Austria

**Keywords:** Lithobiomorpha, Lithobiidae, epipharynx, hypopharynx, phylogeny, systematics

## Abstract

Morphological characters have been widely used in centipede systematics. Here, we aim to obtain morphological information from the preoral chamber and peristomatic structures of lithobiomorph centipedes, with taxonomic sampling focused on the species-rich genus *Lithobius* Leach, 1814. Towards this goal, we (i) examined the epipharynx and hypopharynx of 32 species belonging to four subgenera of the genus *Lithobius*, viz. *Lithobius* Leach, 1814, *Monotarsobius* Verhoeff, 1905, *Sigibius* Chamberlin, 1913 and *Ezembius* Chamberlin, 1919 using light and scanning electron microscopy, (ii) searched for phylogenetically informative characters and (iii) described interspecific variation. Three species of the lithobiid genera *Eupolybothrus* Verhoeff, 1907, *Disphaerobius* Attems, 1926 and *Neolithobius* Stuxberg, 1875 were additionally examined and considered as likely outgroups. New characters and character states are proposed as additions to current phylogenetic datasets. Similarities in the peristomatic structures ally *Disphaerobius* with Lithobius (Ezembius), suggesting that the subfamily Pterygoterginae is nested within Lithobiinae and *Lithobius*.

## Introduction

The peristomatic structures – the epipharynx and hypopharynx – of Chilopoda have hitherto been studied in the orders Scutigeromorpha, Lithobiomorpha, Geophilomorpha (Koch and Edgecombe 2006, 2008, 2012, respectively), and Scolopendromorpha (Edgecombe and Koch 2008, 2009) revealing numerous characters bearing phylogenetically useful information (see also Koch et al. 2010, Vahtera et al. 2013). Two characters of the peristomatic structures, viz. ‘bottle-shaped’ epidermal glandular shafts on the epipharynx and a characteristic shape of the hypopharynx, support the monophyly of the order Lithobiomorpha, whereas paired oblique rows of spines on the clypeal part of the epipharynx are thought to be apomorphic for the family Lithobiidae (Koch and Edgecombe 2008). Until now, *Lithobius*, the most diverse genus in Chilopoda, with more than 500 described species (Zapparoli and Edgecombe 2011, Bonato et al. 2016), is resolved as non-monophyletic on the basis of morphological data. Particular species were recovered in cladistic analysis as most closely related to the genera *Australobius* Chamberlin, 1920, *Hessebius* Verhoeff, 1941, and *Pleurolithobius* Verhoeff, 1899 (Koch and Edgecombe 2008), and this likely applies to other genera of Lithobiinae as well, if not even some of other five subfamilies of Lithobiidae (for current classification of this family see Zapparoli and Edgecombe 2011). However, broad information on species-interrelationships is still missing and the monophyly of subgenera remains questionable, being based on combinations of the same set of characters (Edgecombe 2007). Aiming to obtain further morphological information from the peristomatic structures of *Lithobius* to evaluate whether those might be useful for identifying clades within this very large genus, we study the epipharynx and hypopharynx of 32 species of *Lithobius*, including the subgenera *Lithobius* (23 spp.), *Sigibius* (3 spp.), *Monotarsobius* (5 spp.), and *Ezembius* (1 sp.) using light and scanning electron microscopy. We describe the variation of the microstructures between species and propose new characters for which patterns of variability suggest a potential for phylogenetic analyses. Additionally, we examine species of the lithobiid genera *Neolithobius* Stuxberg, 1875 (Lithobiinae), *Eupolybothrus* Verhoeff, 1907 (Ethopolyinae), and *Disphaerobius* Attems, 1926 (Pterygoterginae), for comparison with *Lithobius*.

## Material and methods

### Material

The studied material consists of 61 specimens belonging to 35 species preserved in 70% or 95% EtOH (Table 1), deposited at the Natural History Museum Vienna (NHMW), the Natural History Museum London (BM/NHMUK) and the Hungarian Natural History Museum Budapest (HNHMB). All material was examined with light and scanning electron microscopy.

**Table 1.** List of studied material deposited in the NHMW, BM/NHMUK and HNHMB.

Species	Studied material
Lithobius (Lithobius) agilis C.L. Koch, 1847	2 females, NHMW 9123, 9124, **Austria**, Niederösterreich, Gaming, F. Feiller leg.
L. (L.) calcaratus C.L. Koch, 1844	1 male, NHMW 9132; 1 female, NHMW 9133, **France**, Normandie, 1919, H. Gadeau de Kerville leg.
L. (L.) carinatus L. Koch, 1862	1 female, NHMW 9125, **Croatia**, Jabuka Island, Pomo, April 1934, F. Werner & O. Wettstein leg.
L. (L.) castaneus Newport, 1844	1 female, NHMW 9194, N36°12'18", E 9°45'35", **Tunisia**, Zaghouan District, Jebel Mansour Mountain, close to (south to) Sidi Aouidette village, pine forest, *Rosmarinus*, under stones and leaf litter, 514 m, 28 March 2008, N. Akkari & P. Stoev leg.
L. (L.) cyrtopus Latzel, 1880	1 female, NHMW 1081, **Poland**, Galizien (früher zu Ungarn), 1919, R. Latzel leg.
L. (L.) dentatus C.L. Koch, 1844	2 females, NHMW 9134, 9135, **Austria**, Wiener Wald
L. (L.) erythrocephalus C.L. Koch, 1847	2 females, NHMW 9136, 9137, **Hungary**, Simontornya, F. Pillich leg.
L. (L.) fagei Demange, 1961	1 male, NHMUK, **Spain**, Majorca, Inca, 1974.242.
L. (L.) forficatus (Linnaeus, 1758)	1 male, NHMW 9138; 1 female, NHMW 9139, **Austria**, Kärnten, Friedlach, 16 October 2001, V. Stagl leg.
L. (L.) lapidicola Meinert, 1872	1 female, NHMW 9196, N 35°32.796' E 11°1.662', **Tunisia**, Mahdia District, Mahdia, touristic area, scattered palm trees and shrubs close to the road, polluted area not far from agricultural land, under stones, 0 m, 16 March 2008, N. Akkari & P. Stoev leg.
L. (L.) latro Meinert, 1872	2 females, NHMW 9140, 9141, **Austria**, Tirol, Zillertal, 1950, Schmölzer leg.
L. (L.) lucifugus L. Koch, 1862	2 females, NHMW 9142, 9143, **Italy**, Südtirol, Sellajoch, 8 August 1896, C. Attems leg.
L. (L.) macilentus L. Koch, 1862	1 male, NHMW 9144, **Austria**, Wien, Niederösterreich, Wiener Wald, 18 December 1892; 25 March 1894; 5 October 1924, C. Attems leg.
L. (L.) mutabilis L. Koch, 1862	2 females, NHMW 9126, 9127, **Czech Republic**, Sudetenländer, 1919, R. Latzel leg.
L. (L.) muticus C.L. Koch, 1847	1 male, NHMW 9145, **Slovenia**, Maribor (Marburg), C. Attems leg.
L. (L.) nodulipes Latzel, 1880	2 females, NHMW 9146, 9147, **Croatia**, Küstenland Kroatien, 1919, R. Latzel leg.
L. (L.) peregrinus Latzel, 1880	1 male, NHMW 9129, **Serbia**, Šar planina mountain range, Ljubeten (=Ljuboten mountain), upper beech forest, 4 June 1906, C. Attems leg.
L. (L.) piceus L. Koch, 1862	1 female, NHMW 9128, **Austria**, österreichische Alpenlande, R. Latzel leg.
L. (L.) pelidnus Haase, 1880	1 male, NHMW 9148, **Austria**, Wiener Wald, Buch leg.
	1male, NHMW 9149, N 48°16'45", E 016°20'10", **Austria**, Wien, 19. Bezirk, Kastralgemeinde Josefsdorf, Kahlenberg Nordosthang, ca. 400 m (Wald, unter Holz), 15 June 1980, J. Gruber leg.
L. (L.) pyrenaicus Meinert, 1872	1 male, NHMW 9130; 1 female, NHMW 9131, **France**, Pyrénées-Orientales, J. Chalande leg.
L. (L.) tenebrosus Meinert, 1872	2 females, NHMW 9151, 9152, **Austria**, Kärnten, Bezirk Sankt Veit an der Glan, Gemeinde Hüttenberg, Pressen (mountain)
L. (L.) tricuspis Meinert, 1872	2 females, NHMW 9153, 9154, **Austria**, Steiermark, Graz, Platte
L. (L.) validus Meinert, 1872	1 female, NHMW 9150, **Austria**, Steiermark, Weiz, Weizenklamm, 1948, H. Franz leg.
L. (Monotarsobius) aeruginosus L. Koch, 1862	2 females, NHMW 7546, **Austria**, Steiermark, Bezirk Liezen, Admont, Kemmatgraben, 1949, Franz H. leg.
	1 male, HNHMB 5980, **Hungary**, Felsőszölnök, Hármasfok, beech-hornbeam forest, 04 August 1948, I. Loksa leg.
L. (M.) austriacus (Verhoeff, 1937)	2 males, HNHMB 5983, 5984, **Hungary**, Salgóbánya, next to Hotel Medves, oak-beech forest, 30 March 2003, L. Dányi leg.
L. (M.) crassipes L. Koch, 1862	2 females, NHMW 9157, 9158, **Germany**, Leipzig, Sturany leg.
	2 females, HNHMB 5981, 5982, **Hungary**, Abaliget, Török-pince Cave (in a forest), at 8 m from the entrance, 14 January 2012, D. Angyal & L. Dányi leg.
L. (M.) curtipes C.L. Koch, 1847	1 female, HNHMB 5985; 1 male, HNHMB 5986, **Hungary**, Győrzámoly, under a woodstem at the side of the dam, 05 October 2000, L. Dányi, Z. Korsós & A. Seres leg.
L. (M.) franciscorum Dányi & Tuf, 2012	2 males, HNHMB 5987, 5988, **Kazakhstan**, Altai Mts., Arshaty, wood near village, 1200 m a.s.l., 30 June 2007, I.H. Tuf leg.
L. (Sigibius) burzenlandicus Verhoeff, 1931	2 males, HNHMB 5989, 5990, N 47°53.456', E 24°31.089', **Romania**, Maramureş Mts, Poienile de Sub Munte, Socolǎu valley, mixed forest, 825m a.s.l., 24 May 2007, Cs. Csuzdi, L. Dányi, J. Kontschán & D. Murányi leg.
L. (S.) microps Meinert, 1868	1 female, 1 male, NHMW 7413, **Hungary**, Siebenbürgen, 1919, R. Latzel leg.
	1 female, HNHMB 5991; 1 male, HNHMB 5992, N 46.1586°, E 8.8804°, **Switzerland**, Magadino, Bolle di Magadino, 195m, under *Reynoutria japonica*, pitfall trap, 2005-2006, M. Moretti leg.
L. (S.) trebinjanus Verhoeff, 1900	1 male, NHMW 9155; 1 female, NHMW 9156, **Albania**, Kukes county/Qarku i Kukësit, Has district/Rrethi i Hasit, Pashtrik mountain range/Mali i Pashtrikut, 1900 m, 1918, A. Penther leg.
L. (Ezembius) electus Silvestri, 1935	1 female, NHMUK, **China**, Kara-Korum, Aghill Dabam (Pass), 4700-4800 m, 30 August 1988, P. Beron leg.
*Neolithobius aztecus* (Humbert & Saussure, 1869)	1 female, NHMUK, BM1894.4.1.75-77, **Guatemala**, Dr. Stoll leg.
*Disphaerobius loricatus* (Sseliwanoff, 1881)	1 male, NHMW 9204, **Kazakhstan**, East-Kazakhstan Area, Kaigutty River Valley, 32 km NW Ayagos, Saline-lend, 15 April 2016, A.A. Fomichev, R.Yu. Dudko leg.
Eupolybothrus (Eupolybothrus) grossipes (C.L. Koch, 1847)	1 male, NHMW 9176, N 46.4916°, E 14.3488°, **Austria**, Kärnten, Bezirk Klagenfurt-Land, Gemeinde Ferlach, Katastralgemeinde Waidisch, 602 m, rocky beech forest with spruce, under stones, logs and from leaf litter, 25 June 2017, Akkari N., Ganske A.-S. & Dányi L. leg.

### Sample preparation

The epipharynx and hypopharynx were dissected from the preoral chamber as described in Koch and Edgecombe (2008) in one to four adult male or female individuals per species. Multifocus images of the sclerotized parts of the epipharynx and hypopharynx were obtained with a Nikon SMZ25 stereomicroscope equipped with a Nikon DS-F2.5 camera using NIS-Elements Microscope Imaging Software with an Extended Depth of Focus (EDF) patch. For scanning electron microscopy (SEM), the specimens were: (1) cleaned in an ultrasonic bath (50–60 Hz) for 5 to 10 seconds (maximum), occasionally in a solution of 15% hydrogen peroxide for 2 hours; (2) dehydrated in an ascending alcohol series (70%, 80%, 90%, 96% EtOH, 2 × 10-15 min each); (3) air dried overnight (or covered with HMDS) or critical point dried (Leica 300 CPD). Specimens were mounted on aluminium stubs equipped with a sticky aluminium tape, glued with conductive silver, coated with platinum (Leica EM SCD500) and studied with a JEOL JSM 6610-LV at an accelerating voltage of 15 kV. Figures were processed with Adobe Photoshop CS6 and assembled in Adobe InDesign CS6.

Terminology follows Koch and Edgecombe (2008).

### List of abbreviations

bdb – labral bristles on distal bar; blf – labral bristles on lateral flap; bsc – ‘button-shaped’ sensilla; bu – single transverse bulge; bud – distal transverse bulge; bup – proximal transverse bulge; db – distal bar; gl – ‘bottle-shaped’ epidermal glandular shafts; hb – hypopharyngeal bar; hsp – hypopharyngeal spine field; lf – lateral flap; lsp – lateral spine field; lmc – paired lips forming median crest; mo – mouth opening; msc – median sensilla cluster; msp – median spine field; nsc – cluster of ‘nipple-shaped’ sensilla; pb – proximal bar; pp – pharyngeal plate; smc – spines flanking median crest; tu – tuft of bristles; tub – tubercles on distal bar; vlb – ventrolateral bar.

## Results

**Epipharynx**

**Figure 1.**
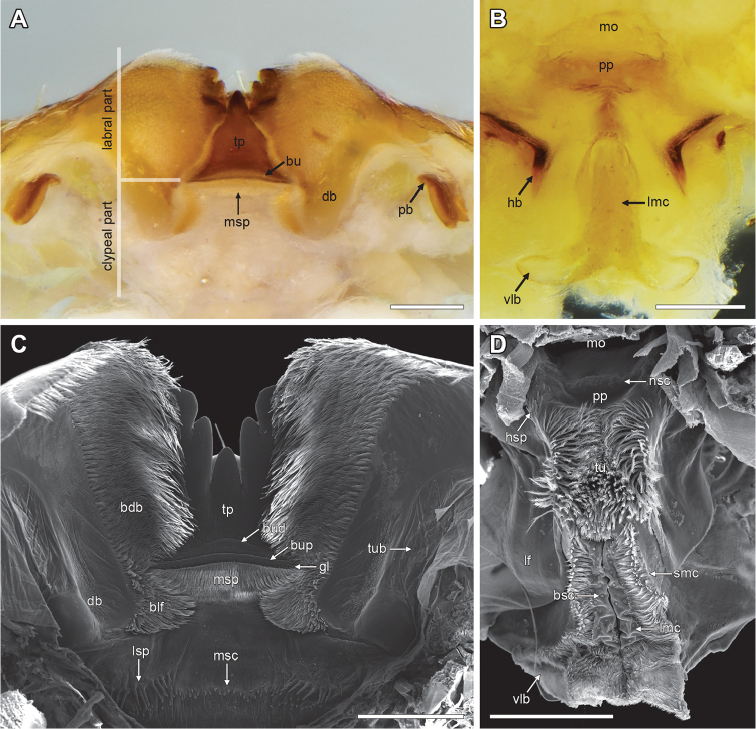
Multifocus light-micrographs and SEM-photographs of peristomatic structures in Lithobiidae. **A** Epipharynx of Lithobius (Lithobius) validus; posterior view (top is ventral) **B** Hypopharynx of Lithobius (Lithobius) carinatus; anterior view (top is dorsal) **C** Epipharynx of Eupolybothrus (Eupolybothrus) grossipes; posterior view (top is ventral) **D** Hypopharynx of Lithobius (Lithobius) forficatus; anterodorsal view (left ventrolateral bar broken). bdb – labral bristles on distal bar, blf – labral bristles on labral flap, bsc – ‘button-shaped’ sensilla, bu – single transverse bulge, bud – distal transverse bulge, bup – proximal transverse bulge, db – distal bar, gl – ‘bottle-shaped’ epidermal glandular shafts, hb – hypopharyngeal bar, hsp – hypopharyngeal spine field, lf – lateral flap, lsp – lateral spine field, lmc – paired lips forming median crest, mo – mouth opening, msc – median sensilla cluster, msp – median spine field, nsc – cluster of ‘nipple-shaped’ sensilla, pb – proximal bar, pp – pharyngeal plate, smc – spines flanking median crest, tp – tooth plate, tu – tuft of bristles, tub – tubercles on distal bar, vlb – ventrolateral bar. Scale bars: 200 µm.

**Figure 2.**
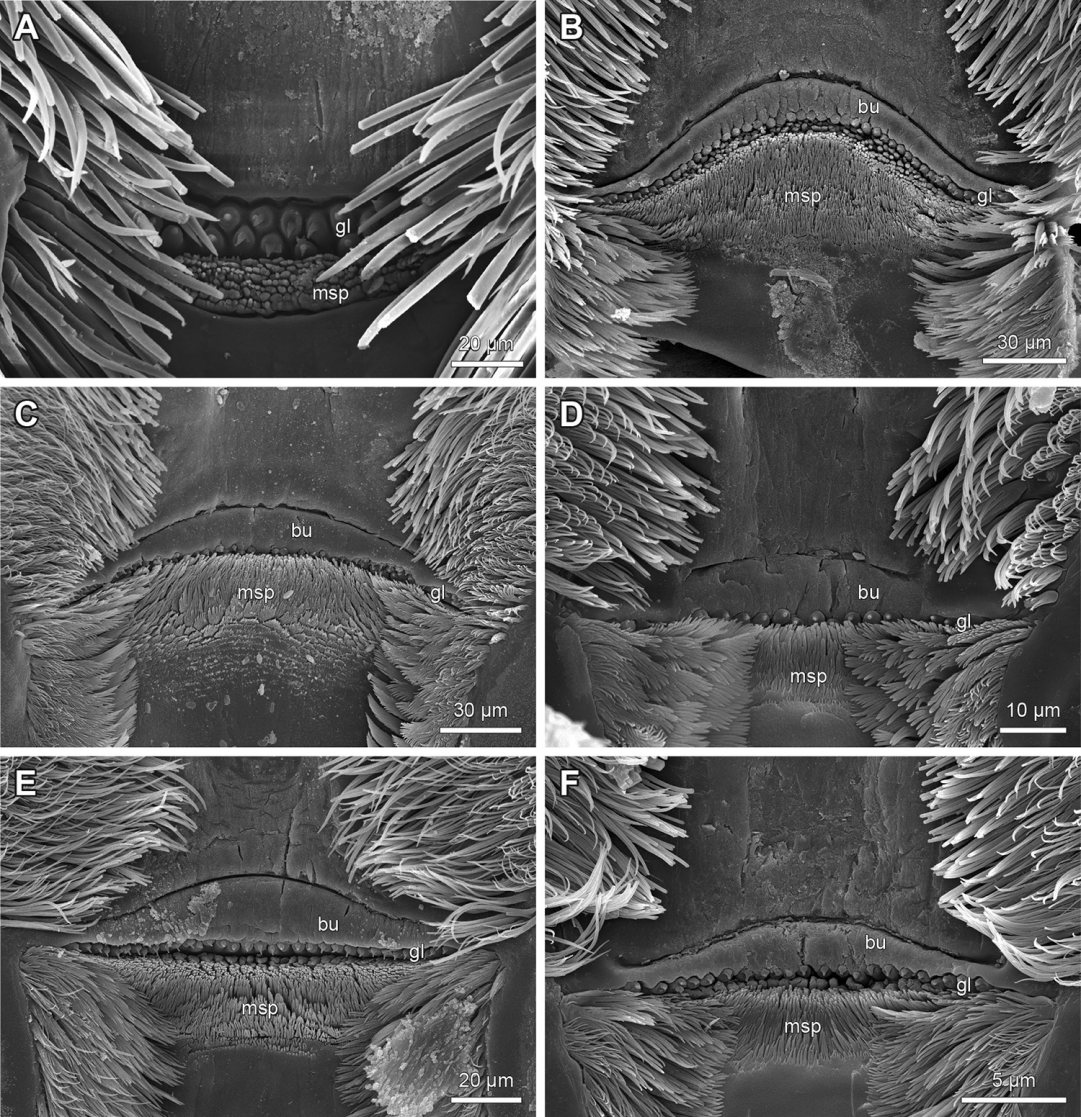
Details of transverse bulge, ‘bottle-shaped’ epidermal glandular shafts and median spine field of the epipharynx of Lithobiidae. **A** *Disphaerobius loricatus*; no transverse bulge; consistently two rows of ‘bottle-shaped’ epidermal glandular shafts; narrow and slightly medially widening median spine field **B** Lithobius (Lithobius) pyrenaicus; parallel aligned margins of a single transverse bulge; one row of ‘bottle-shaped’ epidermal glandular shafts; rhomboid and medially widening median spine field **C** Lithobius (Lithobius) fagei; single transverse bulge with parallel margins; more than one row of ‘bottle-shaped’ epidermal glandular shafts laterally; laterally widening median spine field **D** Lithobius (Sigibius) microps; single transverse bulge with non-parallel margins; subequal width of median spine field **E** Lithobius (Lithobius) mutabilis; single transverse bulge with non-parallel margins; one row of ‘bottle-shaped’ epidermal glandular shafts; subequal width of median spine field **F** Lithobius (Monotarsobius) aeruginosus; single transverse bulge with non-parallel margins; one row of ‘bottle-shaped’ epidermal glandular shafts; subequal width of median spine field. bu – transverse bulge, gl – ‘bottle-shaped’ epidermal glandular shafts, msp – median spine field.

**Figure 3.**
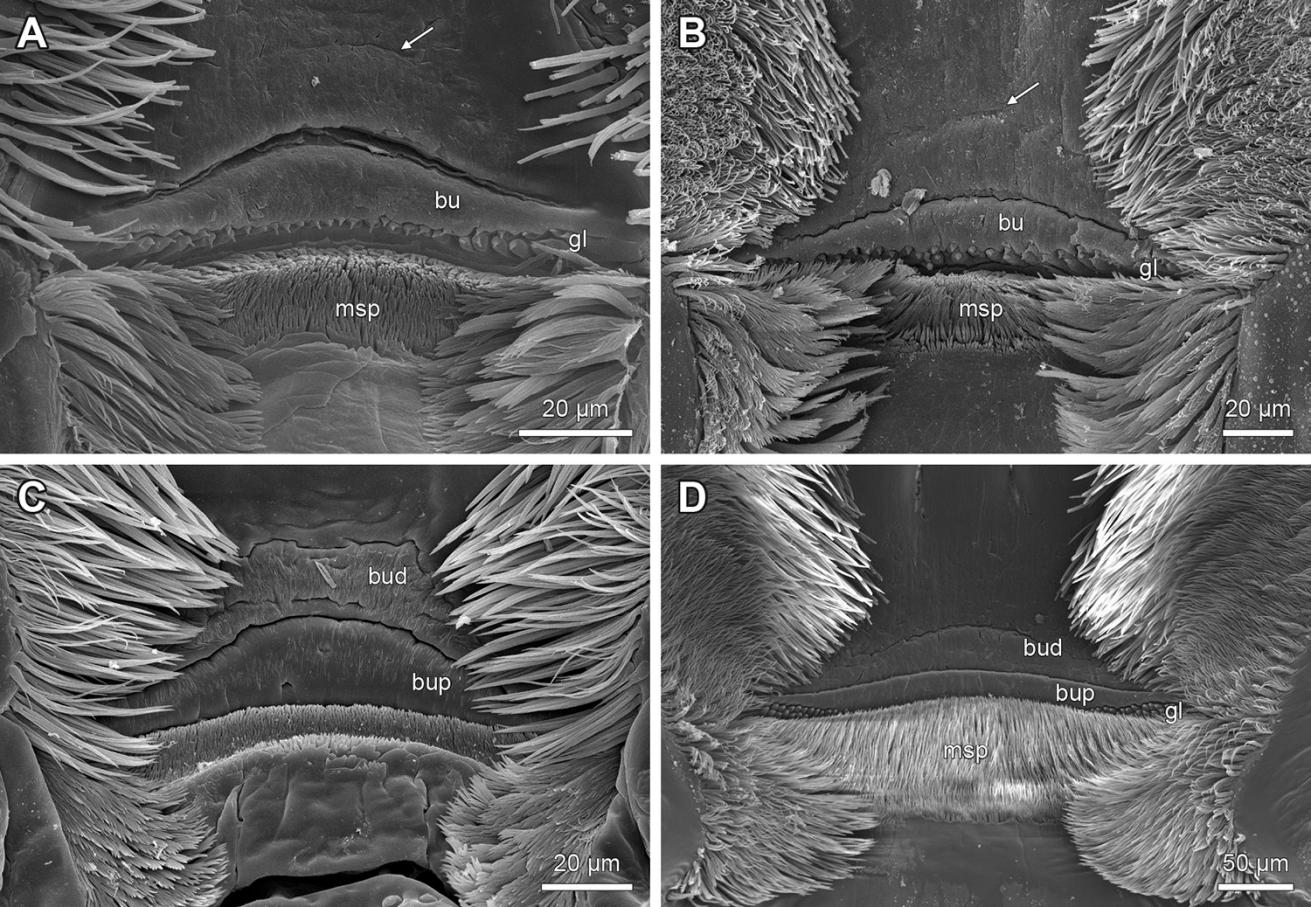
Details of transverse bulge, ‘bottle-shaped’ epidermal glandular shafts and median spine field of the epipharynx of Lithobiidae. **A** Lithobius (Lithobius) macilentus; single transverse bulge with smooth surface (see Fig. 4A) and non-parallel aligned margins; one row of ‘bottle-shaped’ epidermal glandular shafts medially with a transition to two rows laterally (see Fig. 4A); weak transverse furrow distally to the transverse bulge (arrow); laterally widening median spine field **B** Lithobius (Lithobius) piceus; weak transverse furrow (arrow) distally to the single transverse bulge (non-parallel margins); irregular two rows of ‘bottle-shaped’ epidermal glandular shafts; subequal width medially and laterally of median spine field **C** Lithobius (Lithobius) lucifugus; distal and proximal transverse bulges with surface striation (see Fig. 4B–C) **D** Eupolybothrus (Eupolybothrus) grossipes; distal and proximal transverse bulges; medially widening median spine field. bu – transverse bulge, bud – distal transverse bulge, bup – proximal transverse bulge, gl – ‘bottle-shaped’ epidermal glandular shafts, msp – median spine field.

**Figure 4.**
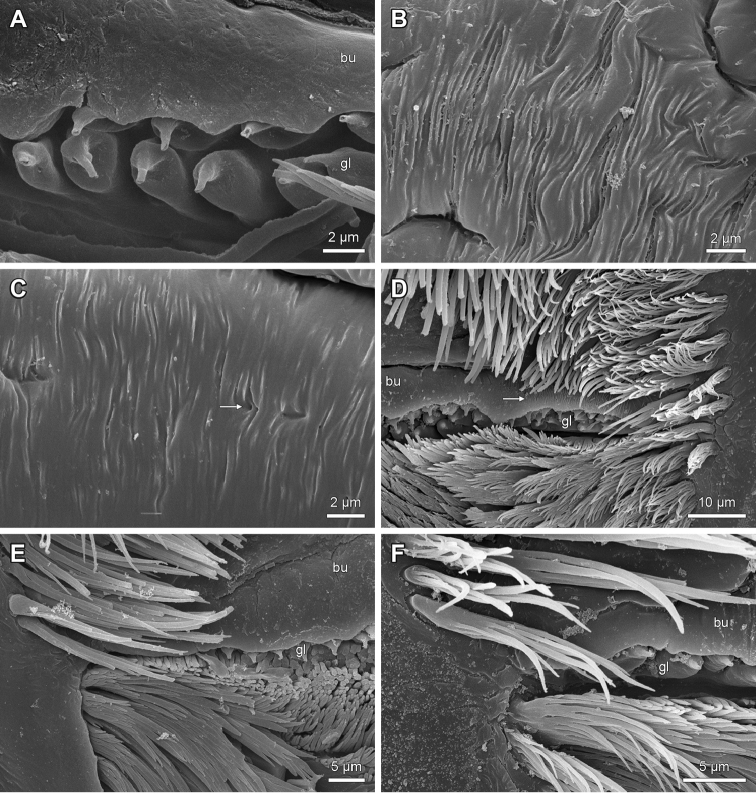
Epipharyngeal structures of *Lithobius*. **A** Lithobius (Lithobius) macilentus; transverse bulge with a smooth surface; two rows of epidermal glandular shafts at the lateral border of the transverse bulge **B** Lithobius (Lithobius) lucifugus; distal transverse bulge with longitudinal striae **C** Lithobius (Lithobius) lucifugus; proximal transverse bulge with longitudinal striae and pores (arrow) **D** Lithobius (Lithobius) fagei; longitudinal striae on the lateral part of the transverse bulge (arrow); continuous branching bristle band from the distal bar to the lateral flap at the margin of the transverse bulge **E** Lithobius (Lithobius) cyrtopus; distinct break of branching bristle band from the distal bar to the lateral flap **F** Lithobius (Monotarsobius) crassipes; distinct break of branching bristle band from the distal bar to the lateral flap. bu – transverse bulge, gl – ‘bottle-shaped’ epidermal glandular shafts.

**Figure 5.**
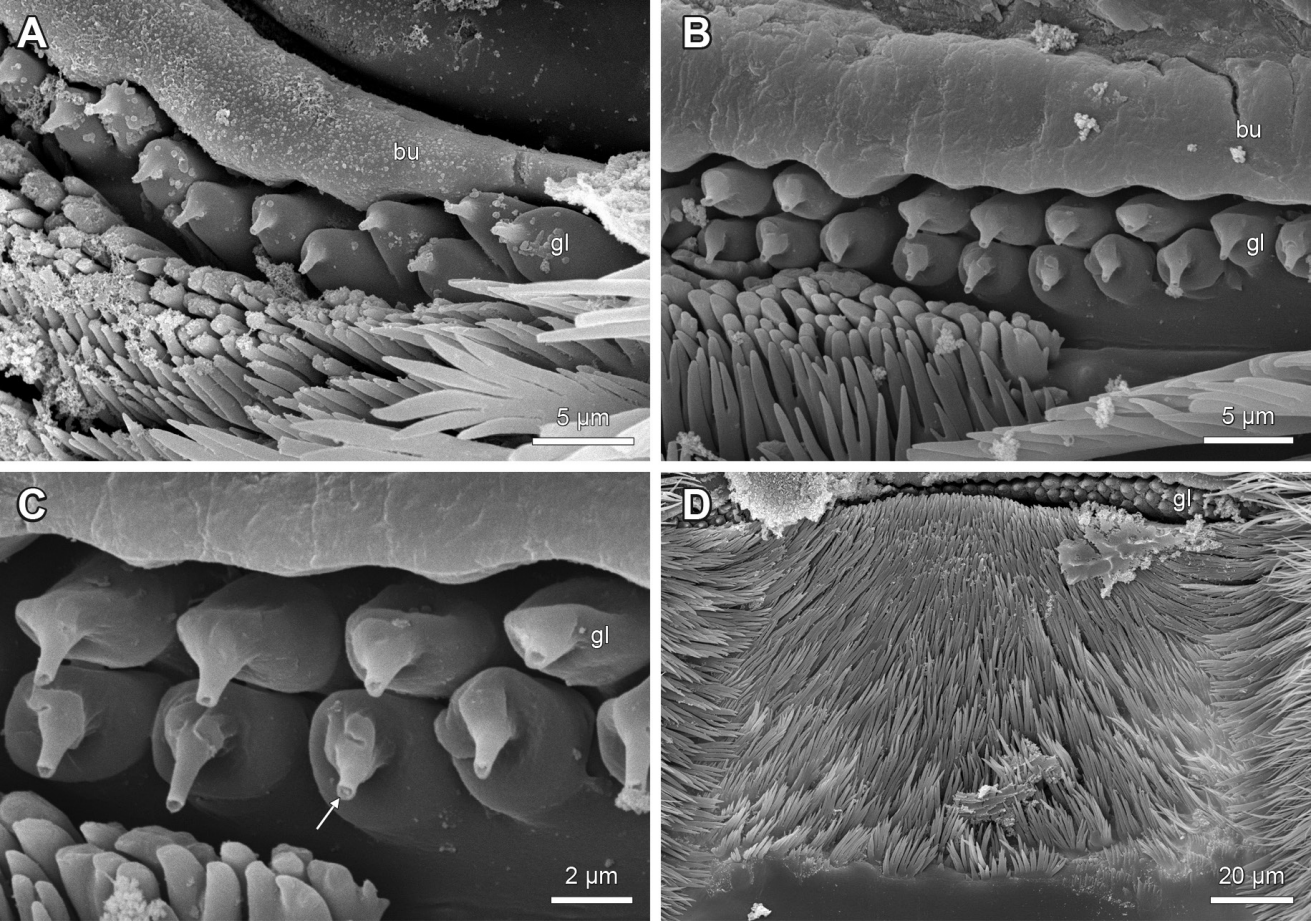
Epipharyngeal structures of *Lithobius*. **A** Lithobius (Lithobius) pyrenaicus; one row of ‘bottle-shaped’ epidermal glandular shafts; laterally narrowing transverse bulge **B–C** Lithobius (Lithobius) validus **B** two rows of ‘bottle-shaped’ epidermal glandular shafts **C** pore of an epidermal glandular shaft (arrow) **D** Lithobius (Ezembius) electus; broad median spine field with several rows of branching bristles and a subequal width medially and laterally. bu – transverse bulge, gl – ‘bottle-shaped’ epidermal glandular shafts.

The epipharynx is distally and proximally bordered by the inner walls of the labrum and the clypeus, respectively (Fig. 1A). Except for *D. loricatus* (Fig. 2A), the labral and clypeal parts of the epipharynx are generally divided by one or two transverse bulges (distal and proximal transverse bulge) (Figs 1A, C, 2B–F, 3: bu, bud, bup). The transverse bulge occurs with a stronger or less pronounced curvature of the furrowed distal and proximal margins bordering the ‘bottle-shaped’ epidermal glandular shafts (Figs 1C: gl, 2B–F, 3A–B). The margins can be parallel or not, curved distally and proximally (Fig. 2B–C) or curved distally and straight proximally (Figs 2D–F, 3A–B). The bulge always narrows laterally (Figs 2B–F, 3A–B, D, 4D–F, 5A). The surface of the bulge(s) is generally smooth (Figs 3A, 4A) but in some species it may show longitudinal striae laterally (Fig. 4D). In *L. tenebrosus* and *L. lucifugus*, the surface of the bulges is longitudinally striated and shows scattered pores (Figs 3C, 4B–C). In other species, a weak transverse furrow occurs on the tooth plate distally to the transverse bulge (Fig. 3A–B).

‘Bottle-shaped’ epidermal glandular shafts always occur proximal to the transverse bulge (Fig. 1C: gl). They can be arranged in one row (Figs 2B, E–F, 5A), one row medially with up to two or more rows on the lateral sides (Figs 2C, 3A, 4A, 5B), or consistently two to more rows (Figs 2A, 3B). The number of glandular shafts varies from 19 in *L. microps* to more than 80 in *L. validus* and is generally higher in larger species. The number of glandular shafts can also differ between individuals of the same species, e.g. 20–22 in *L. aeruginosus* or 42–48 in *L. pyrenaicus*.

**Figure 6.**
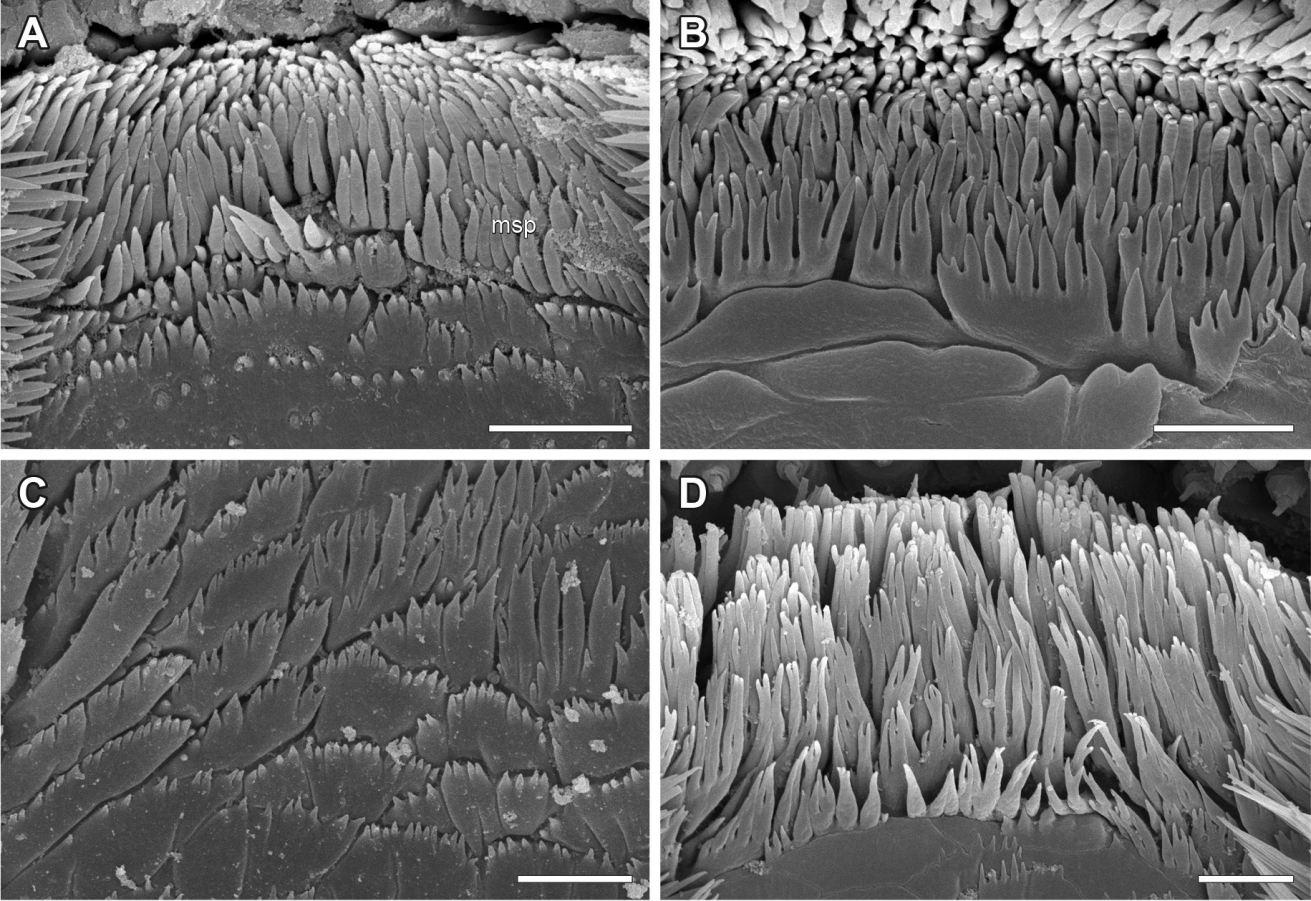
Details of spines from the median spine field on the epipharynx of *Lithobius*. **A** Lithobius (Monotarsobius) aeruginosus; narrow median spine field with a few rows of branching spines **B** Lithobius (Lithobius) macilentus **C** Lithobius (Lithobius) peregrinus **D** Lithobius (Lithobius) tricuspis. msp – median spine field. Scale bars: 5 µm.

Proximal to the ‘bottle-shaped’ epidermal glandular shafts is a median spine field arranged as a wide or a narrow band with a subequal width, medially or laterally widened and consisting of a variable number of branching spines (Figs 1A, C, 2, 3A–B, D: msp, 5D, 6A, D). The spines are always directed towards the labral part of the epipharynx but differ in shape, size and texture. The shape can be scaly, apically furcated or not (Figs 5D, 6).

**Figure 7.**
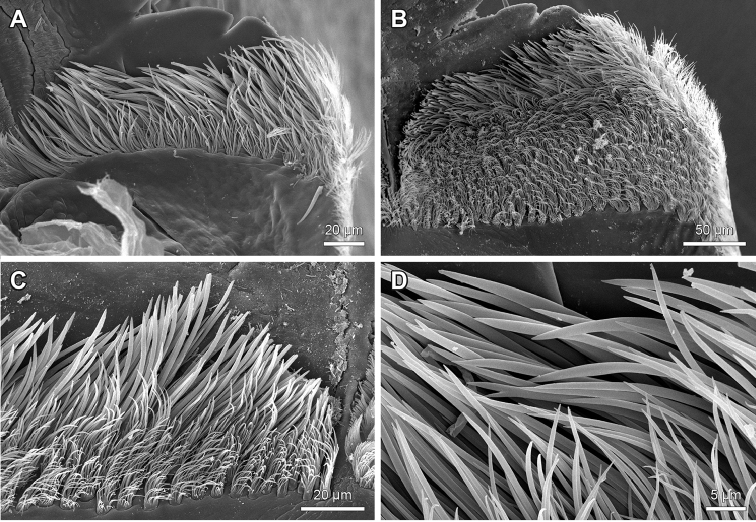
Labral bristle bands on the distal bar of the epipharynx of *Lithobius*. **A** Lithobius (Lithobius) lucifugus; transition of simple to branching bristles from medial to lateral with a few rows of branching bristles **B** Lithobius (Lithobius) peregrinus; transition of simple to branching bristles from medial to lateral with several rows of branching bristles **C** Lithobius (Lithobius) erythrocephalus; detail of the transition of simple to branching bristles from medial to lateral **D** Lithobius (Lithobius) lucifugus; simple bristles on the medial part of the distal bar (top is medial).

**Figure 8.**
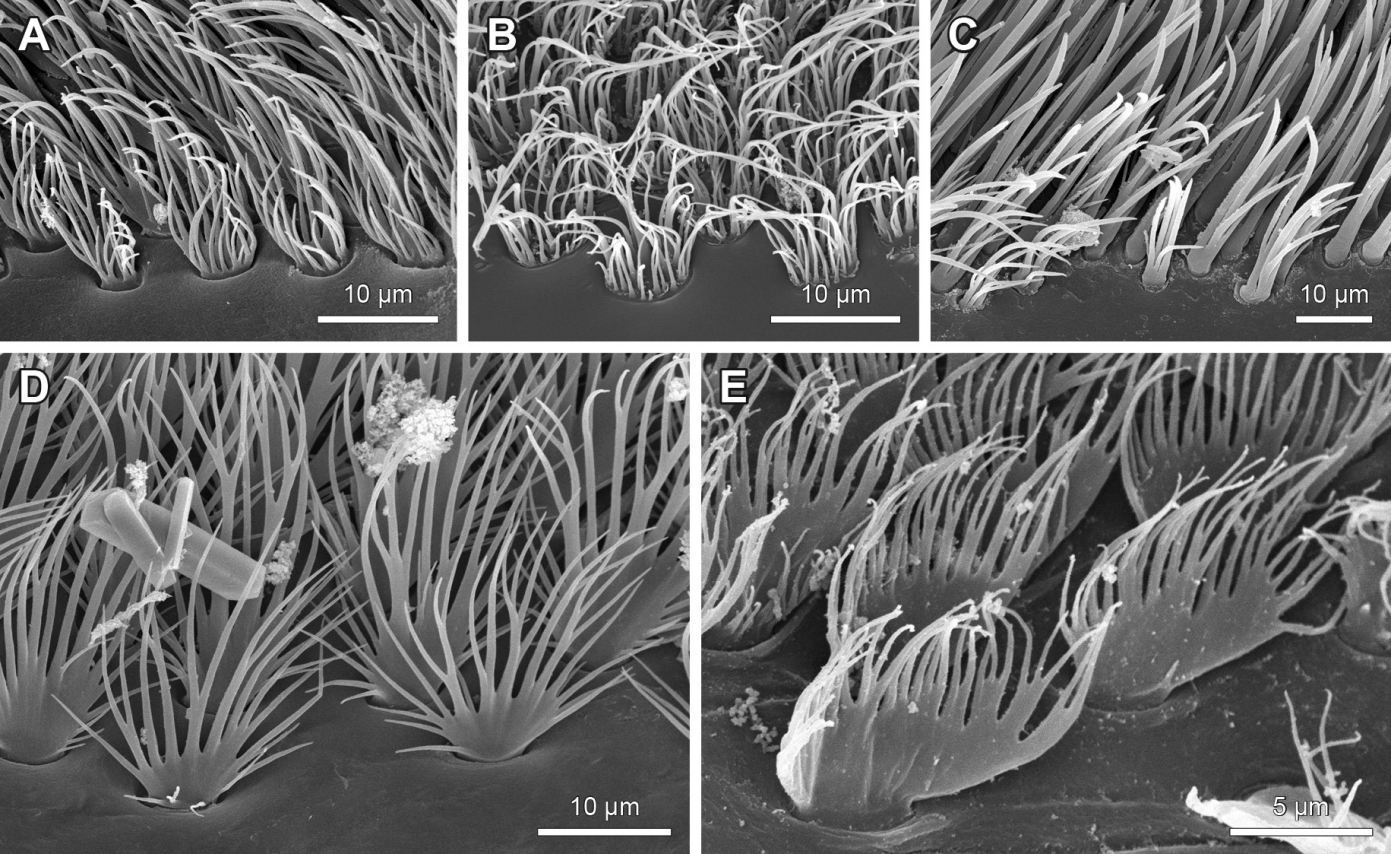
Details of branching bristles on the outer row of the labral bristle bands on the distal bar of the epipharynx of *Lithobius*. **A–B** ‘hassock-like’ branching bristles with a broad base **A** Lithobius (Lithobius) mutabilis **B** Lithobius (Ezembius) electus **C** Lithobius (Lithobius) pyrenaicus; ‘hassock-like’ branching bristles with a narrow base **D** Lithobius (Lithobius) validus; ‘palmleaf-like’ bristles **E** Lithobius (Sigibius) trebinjanus; ‘comb-like’ bristles (top is medial).

Paired labral bristle bands occur on the distal bars on each side of the tooth plate (Fig. 1C: bdb). The bristle bands consist of long, simple bristles medially with a gradual transition to branching bristles laterally (Fig. 7). The branching bristles occur with a few or several outer rows, more or less covering the distal bar (Fig. 7A–B). The bristles point dorsomediad towards the transverse bulge. The branching bristles on the distal bar of the outer rows are generally ‘hassock-like’ (Fig. 8A–C), but they can also be ‘palmleaf-like’ as for *L. validus* (Fig. 8D) or ‘comb-like’ in *L. trebinjanus* (Fig. 8E). The base of the branching bristles ranges from narrow to wide, with intermediate forms (Fig. 8).

**Figure 9.**
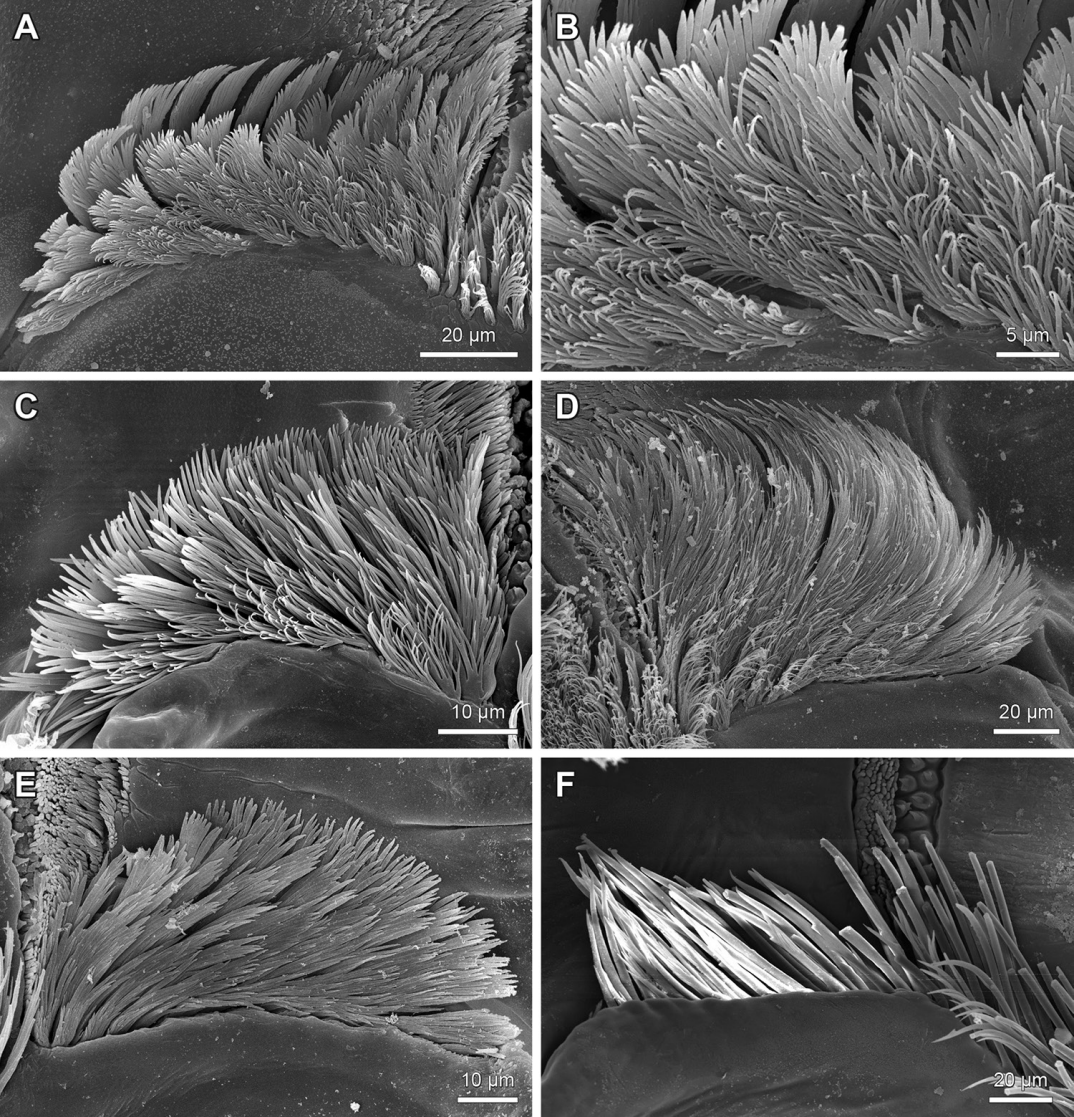
Details of branching bristles on the lateral flap on the distal bar of the epipharynx of *Lithobius*. **A–B** Lithobius (Lithobius) fagei **A** bristles changing from plumose laterally to ‘fan-shaped’ medially **B** detail of 9A **C** Lithobius (Monotarsobius) aeruginosus; bristles changing from plumose laterally to ‘fan-shaped’ medially **D** Lithobius (Lithobius) peregrinus; plumose bristles only **E** Lithobius (Lithobius) cyrtopus; ‘fan-shaped’ bristles only **F** *Disphaerobius loricatus*; simple bristles only (top is medial).

The labral branching bristles on the distal bar expand towards the proximal part in a continuous manner (Fig. 4D) or with a distinct break (Fig. 4E–F) across the transverse bulge to the labral flap margins (Fig. 1C: blf). On the lateral flap, the structure of labral bristles changes gradually from laterally plumose to medially ‘fan-shaped’ (Fig. 9A–C) or it is consistently plumose (Fig. 9D), ‘fan-shaped’ only (Fig. 9E), or they can show just as simple bristles (Fig. 9F).

On the lateral borders of the distal bar, ovoid tubercles are observed in nearly all investigated species (Figs 1C: tub, 10H).

**Figure 10.**
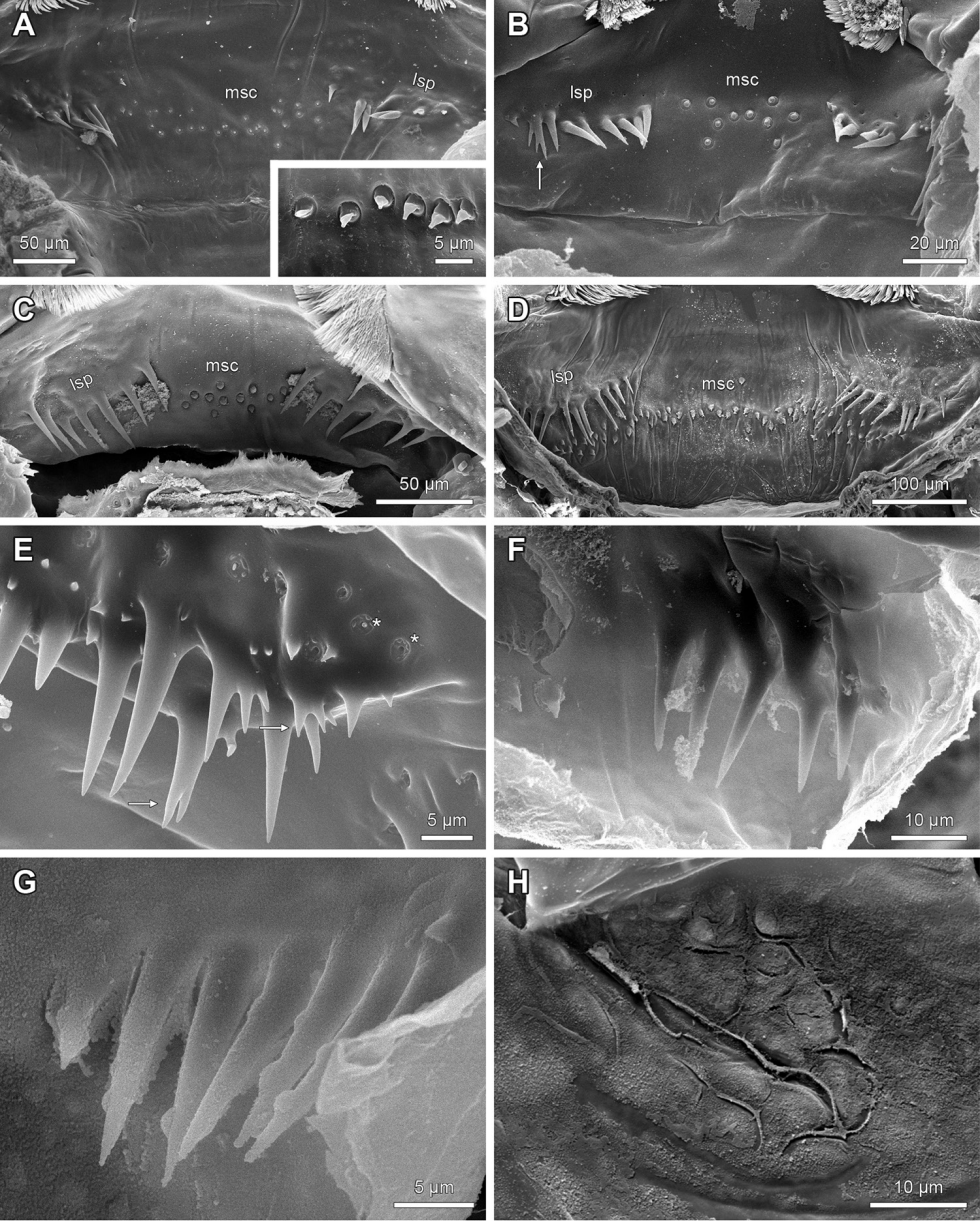
Median sensilla cluster and lateral spine fields on the epipharynx of Lithobiidae. **A** Lithobius (Lithobius) validus; sensilla cluster arranged in an offset pattern; spine field arranged as single oblique row; Inset: Lithobius (Monotarsobius) aeruginosus; sensilla cluster arranged in line **B** Lithobius (Lithobius) tenebrosus; sensilla cluster arranged symmetrically; spine field arranged as single oblique row with trifurcate spines (arrow) **C** *Neolithobius aztecus*; sensilla cluster arranged in an offset pattern; spine field arranged as single oblique row **D** Eupolybothrus (Eupolybothrus) grossipes; sensilla cluster arranged in an offset-pattern and strongly overlapping with lateral spine field proximolaterally; spine field arranged as single oblique row **E** Lithobius (Lithobius) pelidnus; spine field arranged as two rows (tendency of clustering) with bi- or trifurcate spines (arrows) and pores (asterisks) **F** Lithobius (Monotarsobius) curtipes; spine field arranged as single oblique row **G** Lithobius (Monotarsobius) aeruginosus; spine field arranged as single oblique row **H** Lithobius (Monotarsobius) aeruginosus; tubercles on distal bar. lsp – lateral spine field, msc – median sensilla cluster.

The median sensilla cluster (Fig. 1C: msc) on the clypeal part is always transversely aligned. It displays a highly variable interspecific arrangement of the sensilla. These sensilla can be arranged in line (Fig. 10A inset), in an offset-pattern (Fig. 10A, C–D) or symmetrical (Fig. 10B). The number of sensilla in the studied species varies between five in *L. aeruginosus* to 65 in *E. grossipes* (Fig. 10D). Variation of the arrangement and number of sensilla is also recorded in individuals of the same species (e.g. *L. tenebrosus* and *L. aeruginosus*).

Proximal to the clypeal part pairwise lateral spine fields are present bordering the median sensilla cluster except for *N. aztecus* (Fig. 10C), *D. loricatus* and *E. grossipes* (Fig. 10D) in which the sensilla overlap with the spine fields (Fig. 1C: lsp). The lateral spine fields are arranged in one oblique row or more than one row (Fig. 10A-G). If there is more than one row there is a tendency for spines to cluster or form small groups (Fig. 10E). These spines are surrounded by pores (Fig. 10E) and vary in number from two per side in *L. peregrinus* to approximately 17 in *L. crassipes*. They always point proximomediad towards the mouth opening and show a dissimilarity in number and distribution per side within a single individual. The spines are mainly long and tapering, with shorter ones in between (Fig. 10A–G). In some other species, they can be bi- or trifurcate (Fig. 10B, E).

**Hypopharynx**

**Figure 11.**
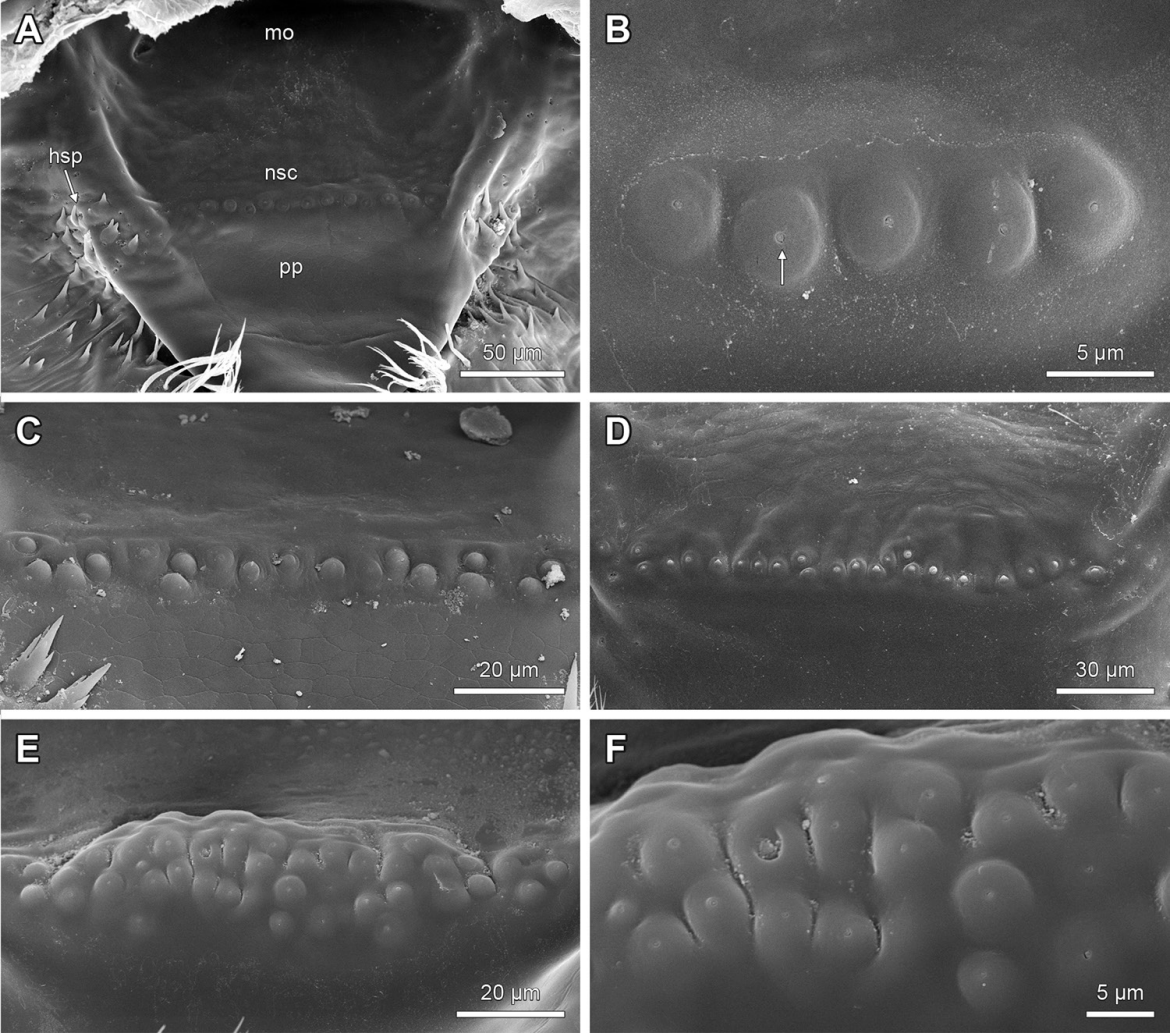
‘Nipple-shaped’ sensilla on pharyngeal plate and hypopharyngeal spines of hypopharynx of *Lithobius*. **A** Lithobius (Lithobius) dentatus; pairwise hypopharyngeal spine fields laterally to pharyngeal plate; transverse line of several ‘nipple-shaped’ sensilla **B** Lithobius (Monotarsobius) aeruginosus; transverse line of a few ‘nipple-shaped’ sensilla on the pharyngeal plate; arrow indicates a pore **C** Lithobius (Lithobius) pyrenaicus; ‘nipple-shaped’ sensilla arranged in a zig-zag-pattern **D** Lithobius (Lithobius) validus; several ‘nipple-shaped’ sensilla arranged in a transverse line with some offset sensilla **E–F** Lithobius (Lithobius) forficatus **E** clustered ‘nipple-shaped’ sensilla **F** high magnification of ‘nipple-shaped’ sensilla from Fig. 11E. hsp – hypopharyngeal spine field, mo – mouth opening, nsc – cluster of ‘nipple-shaped’ sensilla, pp – pharyngeal plate.

The hypopharynx is a subtriangular outgrowth consisting of paired lips forming a median crest (Fig. 1B, D: lmc). In front of the mouth opening lies the pharyngeal plate (*Schlundplatte* after Verhoeff 1902-1925) (Figs 1B, D, 11A: mo, pp). The latter shows transversely arranged ‘nipple-shaped’ sensilla on its median part (Figs 1D, 11A: nsc). The number of these sensilla varies from five in *L. aeruginosus* (Fig. 11B) to 25 in *L. validus* (Fig. 11D). The distribution pattern of ‘nipple-shaped’ sensilla varies from one clear line (Fig. 11A–B), zig-zag (Fig. 11C) to clusters of sensilla (Fig. 11E–F) but also displays intermediate forms (Fig. 11D).

**Figure 12.**
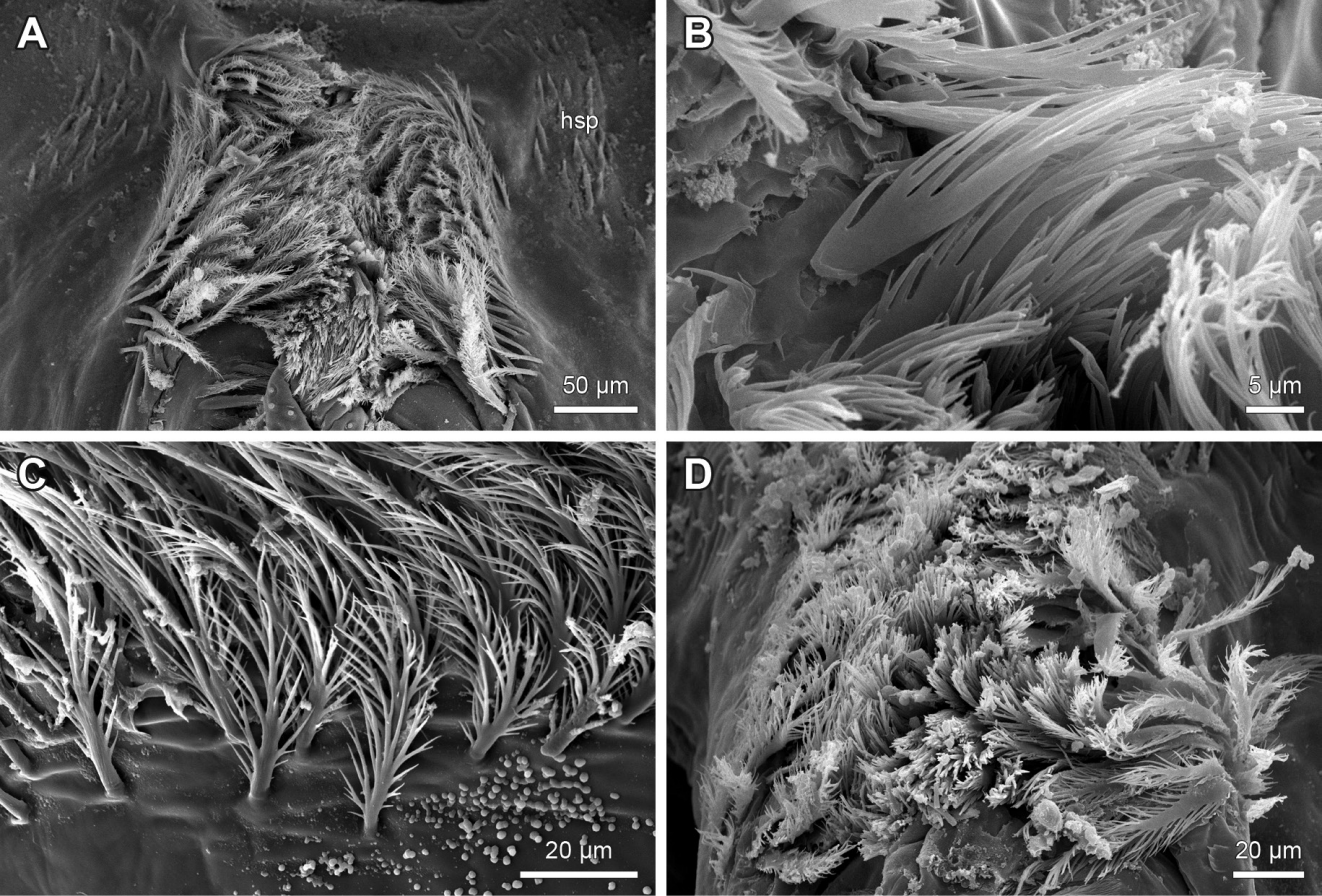
Shapes of branching bristles forming a tuft distally to the pharyngeal plate of the hypopharynx of Lithobiidae. **A** Lithobius (Lithobius) forficatus; ramified branching bristles with a roundish shaft and hypopharyngeal spines laterally to pharyngeal plate (top is dorsal) **B** Lithobius (Lithobius) calcaratus; close-up of ‘fan-shaped’ and flattened branching bristles (top is medial) **C** Eupolybothrus (Eupolybothrus) grossipes; ramified and more flattened branching bristles (top is dorsal) **D** Lithobius (Lithobius) latro; ramified and flat branching bristles (top is medial). hsp – hypopharyngeal spine field.

**Figure 13.**
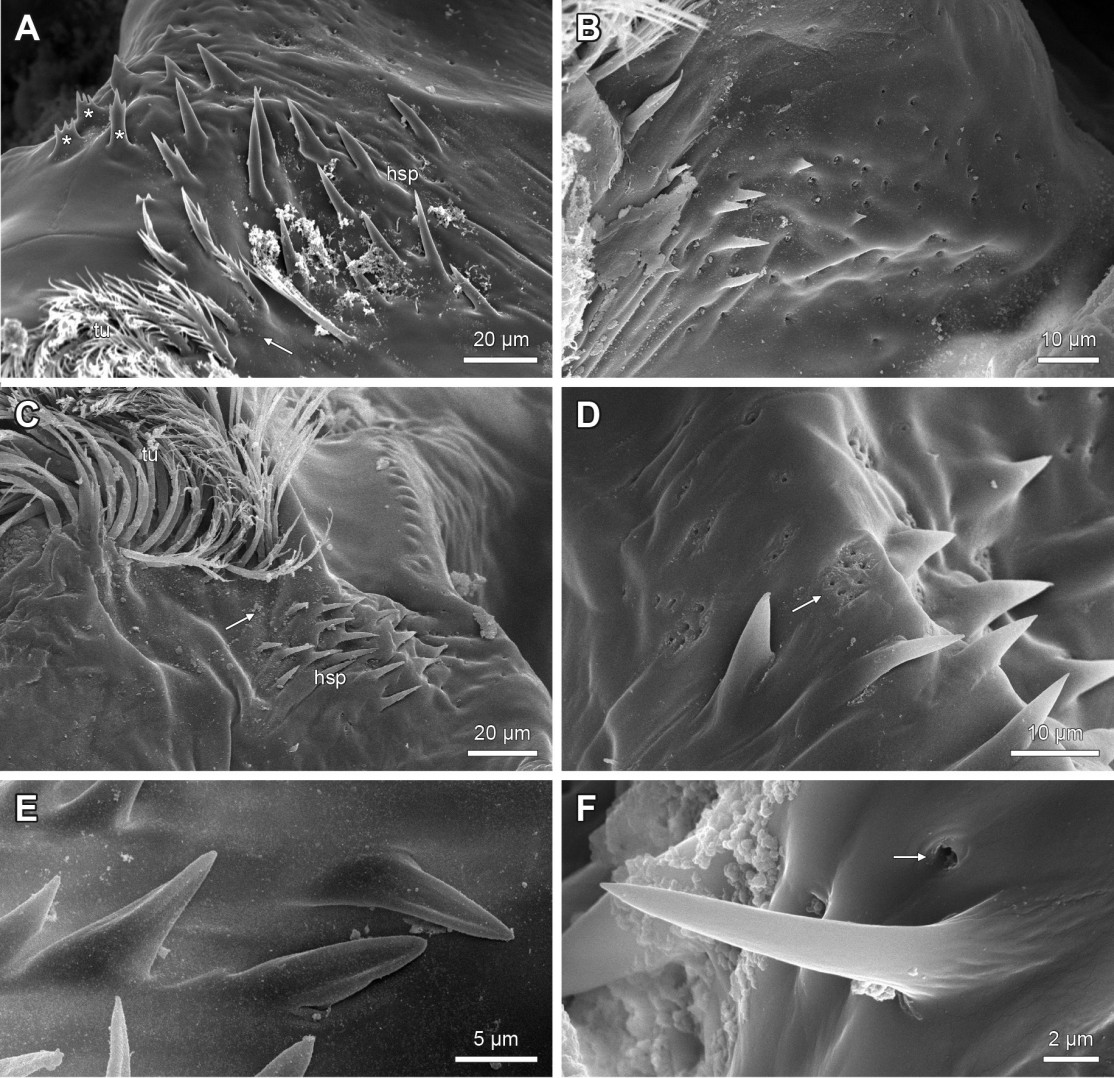
Examples of number and shape of the hypopharyngeal spines and surrounding pores of Lithobiidae. **A** Eupolybothrus (Eupolybothrus) grossipes; several tapering spines with trifurcate spines (asterisks) in between and a continuous transition to the tuft area (arrow) **B** Lithobius (Lithobius) agilis; few short tapering spines; several single pores **C** Lithobius (Lithobius) muticus; long and tapering spines; distinct break (arrow) between hypopharyngeal spine field and branching bristles of tuft **D** Lithobius (Lithobius) validus; hypopharyngeal spines surrounded by cluster of up to six pores (arrow) **E** Lithobius (Lithobius) cyrtopus; flattened and ridged spines **F** Lithobius (Lithobius) castaneus; detail of a long tapering spine close to a single pore (arrow). hsp – hypopharyngeal spine field, tu – tuft of bristles.

Distal to the pharyngeal plate appears a ‘tuft-like’ cluster of branching bristles (Fig. 1D: tu). The shape of these branching bristles varies from ‘fan-shaped’ to ramified, with a more flattened or roundish shaft occurring with several intermediate forms (Figs 12, 13C).

Lateral to the pharyngeal plate, hypopharyngeal spines are always present (Figs 1D, 11A, 12A: hsp). They are arranged in clusters of five to 37 spines unilaterally (Fig. 13A–C) and they are surrounded with single or clustered pores (up to six) from apparently epidermal glands (Fig. 13A–B, D, F). The spines mainly taper (Figs 11A, 13A–D, F), sometimes with ridges along the lateral side of the spine shaft (Fig. 13E) or are apically furcate (Fig. 13A). They can be long or short, sometimes with a more flattened appearance (Figs 11A, 13). The hypopharyngeal spines may occur with a continuous transition distomedially to the tuft area (Fig. 13A) or with a distinct break (Fig. 13C).

**Figure 14.**
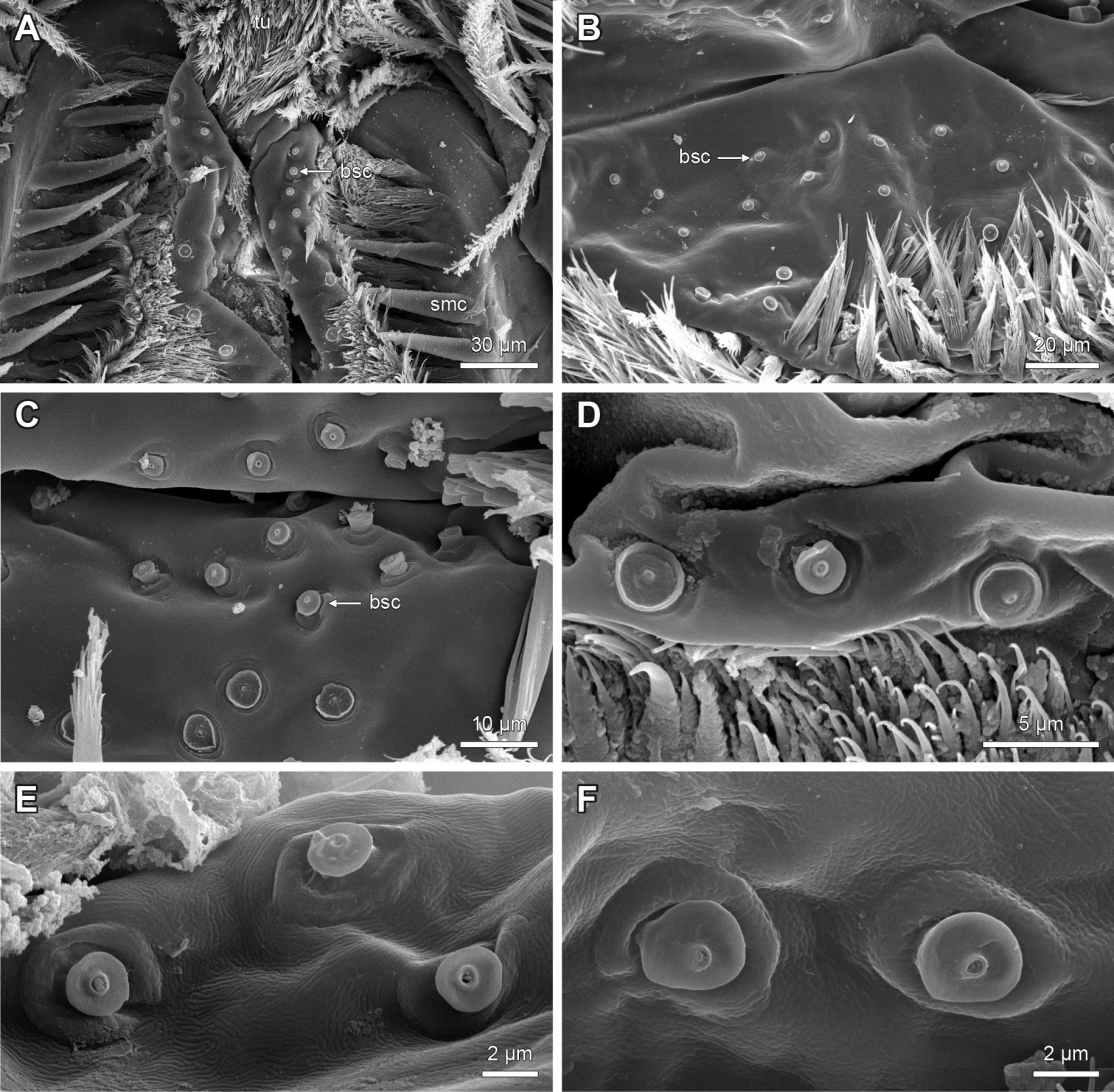
Examples of ‘button-shaped’ sensilla on the lips of hypopharynx of Lithobiidae. **A** Lithobius (Lithobius) forficatus; proximal part of lips forming median crest with cluster of ‘button-shaped’ sensilla; flattened spines flanking median crest margin **B** Eupolybothrus (Eupolybothrus) grossipes; left lip with cluster of ‘button-shaped’ sensilla **C** Lithobius (Lithobius) validus **D** Lithobius (Sigibius) burzenlandicus **E** Lithobius (Lithobius) muticus **F** Lithobius (Lithobius) carinatus. bsc – ‘button-shaped’ sensilla, smc – spines flanking median crest, tu – tuft of bristles. **A** top is dorsal; **B–F** top is medial.

**Figure 15.**
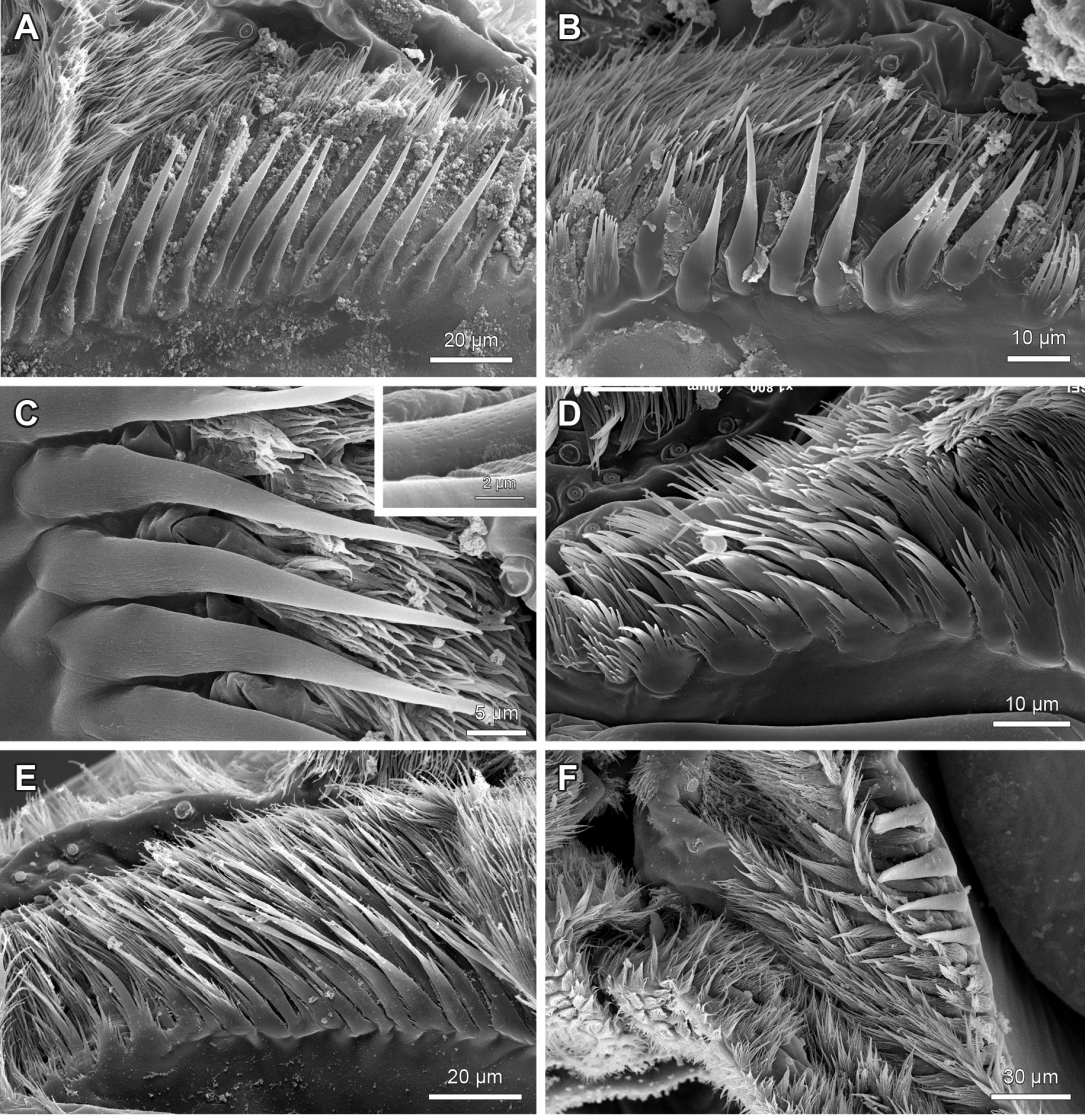
Examples of spines and bristles flanking the median crest margins of hypopharynx of *Lithobius*. **A–C** flattened spines with a transition to branching bristles on the inner rows **A** Lithobius (Lithobius) pelidnus **B** Lithobius (Monotarsobius) franciscorum **C** Lithobius (Lithobius) muticus; Inset: detail of structured surface of flattened spines **D–E** continuously branching bristles flanking the median crest **D** Lithobius (Sigibius) microps; stout and short branching bristles **E** Lithobius (Lithobius) piceus; slender and long branching bristles **F** Lithobius (Lithobius) forficatus; flattened spines flanking median crest margin. **A–B, D–E** top is medial; **C** top is ventral; **F** top is dorsal.

‘Button-shaped’ sensilla are arranged in continuous clusters on the lips of the median crest medially up to the ventrolateral bars within the branching bristles and are present in all examined species (Figs 1D: bsc, 14, 16B–D). The median crest is flanked by intergrading rows of branching bristles (Fig. 1D: smc), which can be stout and short (Fig. 15D) or slender and long (Fig. 15E). In several species, we observed a transition from branching bristles to flattened spines on the outermost rows (Figs 14A, 15A–C, F). The flattened spines show a structured surface (Fig. 15C).

**Figure 16.**
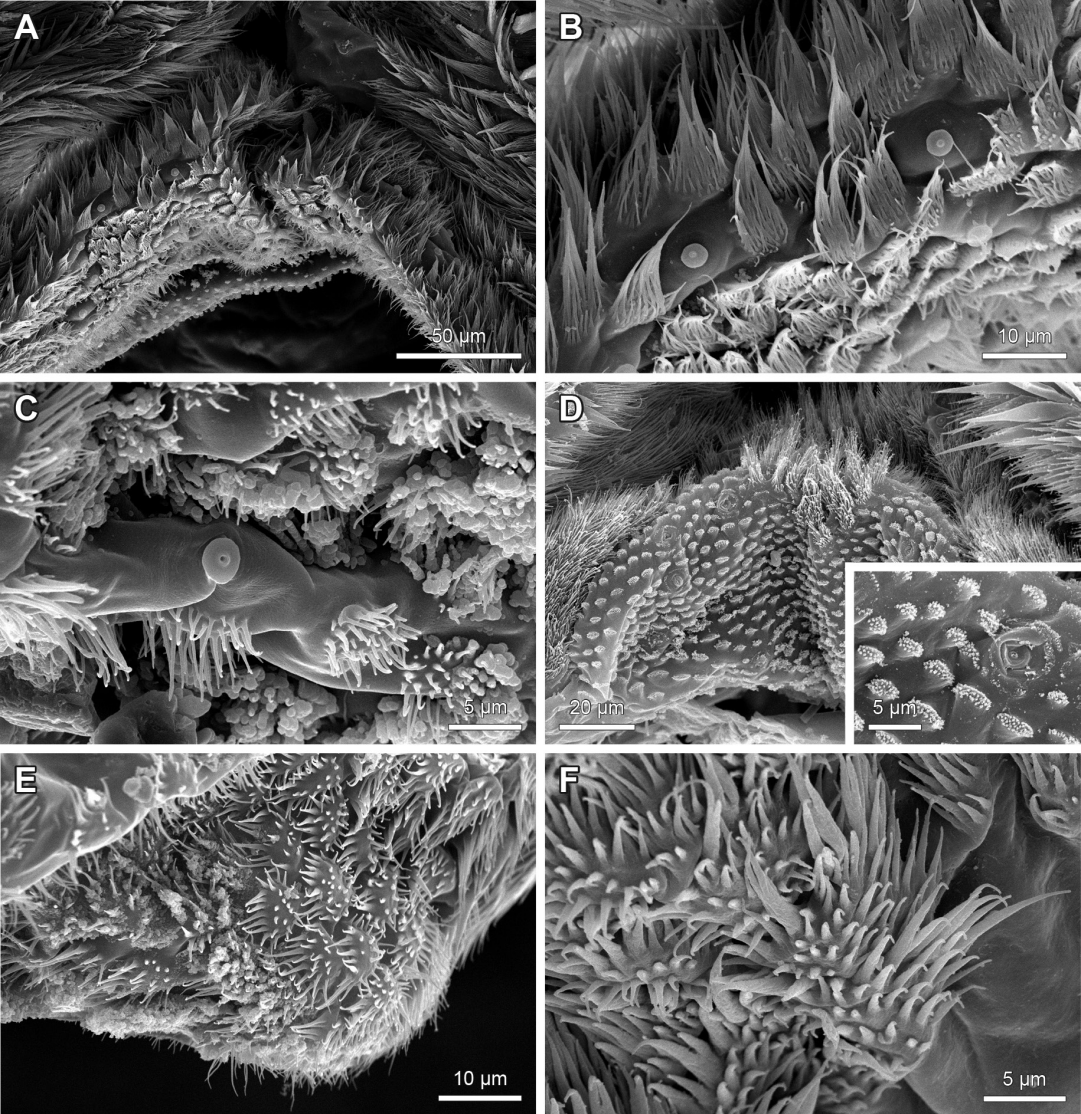
Examples of bristles transitioning in shape and length along the median crest margin on hypopharynx of *Lithobius*. **A–B** Lithobius (Lithobius) forficatus **B** ‘button-shaped’ sensilla between branching bristles on the distal part of the lips **C** Lithobius (Lithobius) pyrenaicus **D** Lithobius (Lithobius) erythrocephalus; Inset: detail of brush-tufts surrounding ‘button-shaped’ sensilla **E** Lithobius (Lithobius) pelidnus **F** Lithobius (Lithobius) carinatus.

**Figure 17.**
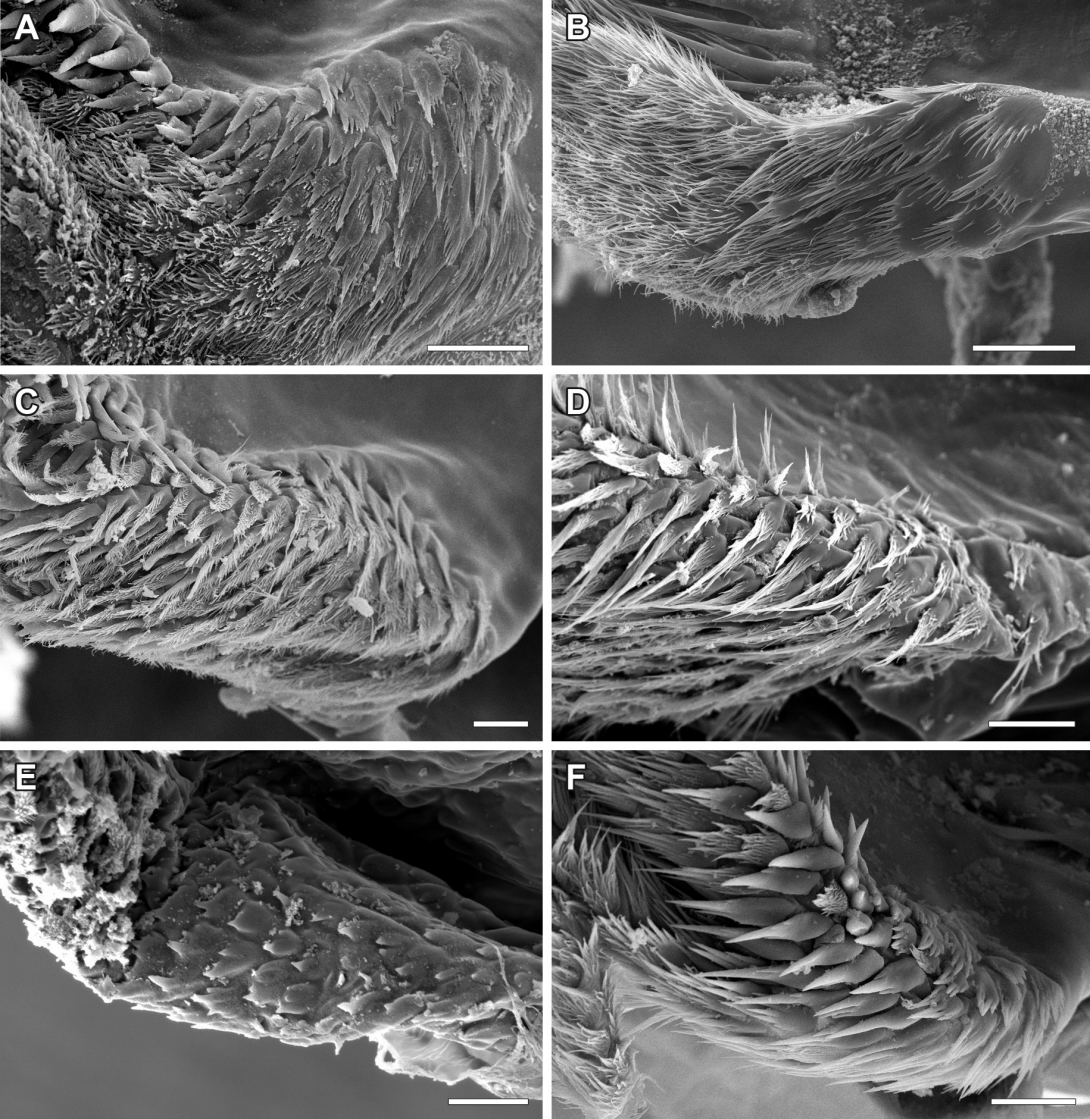
Bristles and scales on the distal tips of the lips on hypopharynx of Lithobiidae. **A** Lithobius (Lithobius) cyrtopus **B** Lithobius (Lithobius) pelidnus **C** Lithobius (Lithobius) validus **D** Eupolybothrus (Eupolybothrus) grossipes **E** *Disphaerobius loricatus*; scales **F** Lithobius (Lithobius) forficatus. Scale bars: 20 µm.

The trichomes on the paired lips forming the median crest exhibit an intergrading transition from the tuft area proximal to distal up to the tips of the ventrolateral bars and medially to the proximoventral parts of the hypopharynx (Fig. 1D). At the border to the tuft area, there are generally ‘fan-shaped’ or plumose branching bristles, which mostly shorten in length, transitioning to ‘brush-’, ‘tuft-’, ‘feather-like’ or simple bristles (Figs 14A, 15A, E–F, 16A–C, E–F, 17A–D, F). On the proximoventral part, the bristles change over into clearly separated brush-tufts that are intermingled by ‘button-shaped’ sensilla (Fig. 16D). The shape of trichomes varies greatly between species. In *D. loricatus*, for example, there are scales on the distal tips of the lips bordered by the margin of the ventrolateral bar (Fig. 17E) in comparison to other species showing bristles in this area (Fig. 17A–D, F).

### Peristomatic characters with phylogenetic significance

In the following, eight peristomatic characters are proposed for the genus *Lithobius*, three of which are newly described (see char. 4, 6, 7). Additionally, we verified the consistency of two characters (see char. 2, 8) and adjusted three (see char. 1, 3, 5) from those indicated by Koch and Edgecombe (2008). Codings are provided in Appendix 1.

**Epipharynx**

1. ‘Bottle-shaped’ glandular shafts at the border between labral and clypeal part of epipharynx: (0) one distinct regular row; (1) more than one regular or irregular row.

All the investigated lithobiomorph species possess ‘bottle-shaped’ epidermal glandular shafts at the border between the labral and clypeal parts of the epipharynx. The latter can be in one regular row (Figs 2B, D–F, 5A) or with a variable arrangement, e.g. one regular row medially, which expands to two or three regular or irregular rows laterally (Figs 2C, 3A, 4A, 5B). A regular or irregular arrangement of consistently two or more rows along the whole width is present, for example, for *D. loricatus* and *L. piceus* (Figs 2A, 3B). Both states were identified across all subgenera of *Lithobius* with state (0) being underrepresented in the subgenus Lithobius (6 of 23 examined species). *N. aztecus*, *D. loricatus* (Fig. 2A) and *E. grossipes* (Fig. 3D) share state (1).

2. Labral bristle bands of epipharynx: (0) bristle bands continuous across transverse bulge; (1) distinct break in bristle bands proximal and distal to transverse bulge. (Character 31 in Koch and Edgecombe 2008).

The subgenera of *Lithobius* and other lithobiid genera show labral bristle bands that are either continuous (Fig. 4D) or are interrupted at the transverse bulge (Fig. 4E–F). All studied species of the subgenus Sigibius share state (1) (e.g. Fig. 2D).

3. Number of transverse bulge(s) at border between labral and clypeal parts of epipharynx: (0) none; (1) one; (2) two.

The presence of one or two transverse bulges is common for the genera *Lithobius*, *Neolithobius* and *Eupolybothrus* (e.g. Figs 2B–F, 3). The bulges are absent only in the genus *Disphaerobius* (Fig. 2A). Two bulges are shared by *L. calcaratus*, *L. lucifugus*, *L. tenebrosus* and *E. grossipes* only (e.g. Fig. 3C–D).

4. Direction of distal and proximal furrowed margins of transverse bulge or transverse bulges on epipharynx: (0) parallel; (1) non-parallel.

Both states occur in all studied genera, state (0) e.g. in *L. pyrenaicus*, *L. fagei*, *L. lucifugus* and *E. grossipes* (Figs 2B–C, 3C–D) or state (1) e.g. in *L. microps*, *L. mutabilis*, *L. aeruginosus*, *L. macilentus* and *L. piceus* (Figs 2D–F, 3A–B). All species of the subgenus Sigibius share a non-parallel alignment (state (1)) of the transverse bulge margins (e.g. *L. microps*; Fig. 2D).

5. Median field of branching spines immediately proximal to the border between labral and clypeal parts of epipharynx: (0) rhomboid, widening medially; (1) widening laterally; (2) subequal width medially and laterally.

State (2) is most common throughout the subgenus Lithobius and occurs in the other subgenera of *Lithobius*, e.g. *L. microps*, *L. piceus* and *L. electus* (Figs 2D, 3B, 5D). The genera *Eupolybothrus*, *Disphaerobius* and *Neolithobius* share state (0) but show variation in the number of rows of branching spines (e.g. Figs 2A, 3D). All states occur with a narrower or wider band having a few or several rows of branching spines.

6. Shape of branching bristles on labral flap of epipharynx: (0) lateral to medial transition from plumose to ‘fan-shaped’ bristles; (1) ‘fan-shaped’ bristles only; (2) plumose bristles only; (3) simple bristles only.

A transition of branching bristles from plumose laterally to ‘fan-shaped’ medially is the most common state (0) across the genus *Lithobius*, and also pertains to *Neolithobius* and *Eupolybothrus* (e.g. Fig. 9A–C). State (1) was observed in *L. cyrtopus* (Fig. 9E), *L. lucifugus*, *L. pelidnus* and *L. microps.* State (2) was present in *L. peregrinus* (Fig. 9D), *L. piceus* and *L. tricuspis*, and state (3) in *D. loricatus* only (Fig. 9F).

7. Lateral expansion of median sensilla cluster of epipharynx: (0) isolated from the lateral spine fields; (1) partly overlapping with the lateral spine fields.

In all *Lithobius* species we examined (except for *L. tricuspis* and *L. nodulipes* for which the samples were damaged), the median sensilla cluster is bordered laterally by fields of spines (state (0); Fig. 10A–B). The sensilla in *D. loricatus* and *N. aztecus* slightly overlap with the lateral spine fields medially (state (1); e.g. Fig. 10C). In *E. grossipes* the sensilla of the median sensilla cluster strongly overlap with the lateral spine fields proximolaterally (state (1); Fig. 10D).

8. Differentiation of spines flanking median crest of hypopharynx: (0) intergrading rows of branching bristles; (1) single outer row of simple flattened spines with abrupt transition to multifurcating inner rows of branching bristles. (Character 39 in Koch and Edgecombe 2008)

Species of the subgenus Monotarsobius always display state (1) (e.g. *L. franciscorum*; Fig. 15B). The *Ezembius* species *L. electus* studied here displays state (1), which differs from Lithobius (Ezembius) giganteus Sseliwanoff, 1881, stated by Koch and Edgecombe (2008). State (1) (Fig. 15A–C, F) is more common throughout the other subgenera of *Lithobius* and species of the other examined genera compared to state (0) (Fig. 15D–E).

## Discussion

Studies on the external morphology and microanatomy of the peristomatic structures of centipedes have hitherto unveiled phylogenetically useful information (Koch and Edgecombe 2006, 2008, 2012, Edgecombe and Koch 2008, 2009). The ‘bottle-shaped’ epidermal glandular shafts of the epipharynx and the discrete shape of the hypopharynx support the monophyly of the order Lithobiomorpha and paired oblique rows of lateral spines on the clypeal part of the epipharynx is, for example, considered as an apomorphic character for the family Lithobiidae (Koch and Edgecombe 2008). The inclusion of characters from these structures in a morphological dataset that also included other (mostly external) parts of the body further revealed the genus *Lithobius* as a non-monophyletic taxon (Koch and Edgecombe 2008). Within the genus *Lithobius*, five out of eleven described characters of the peristomatic structures display different states (Koch and Edgecombe 2008), which might give hints on species-interrelationships within the genus. These data from the peristomatic structures are presented as a set of coded characters (Appendix 1) that will be analysed cladistically with characters from other character systems in a later study.

### Phylogenetic significance of the peristomatic structures of Lithobiidae

While studying the peristomatic structures of Lithobiomorpha and Scutigeromorpha, Koch and Edgecombe (2008) compared the presence of the ‘bottle-shaped’ epidermal glandular shafts between the labral and clypeal part of the epipharynx. These glandular shafts were reported to be constantly present in Lithobiomorpha (Koch and Edgecombe 2008) and absent in other chilopods (Koch and Edgecombe 2006, 2008, 2012, Edgecombe and Koch 2008). We confirmed the presence of glandular shafts in the specimens we examined in the lithobiid genera *Lithobius*, *Neolithobius*, *Eupolybothrus* and *Disphaerobius* and further recorded differences in number and regularity of rows (character 1).

The same authors (Koch and Edgecombe 2008) described the presence of a transverse bulge dividing the labral and clypeal part on the epipharynx for all Lithobiomorpha except for *Hessebius plumatus* Zalesskaja, 1978 and L. (Ezembius) giganteus displaying no bulge at all. This study confirms the absence of the bulge in the species *D. loricatus* (Fig. 2A) and for the first time the presence of a second bulge (distal transverse bulge) as recorded for the species *L. calcaratus*, *L. lucifugus*, *L. tenebrosus* and *E. grossipes* as well as *E. fasciatus* (Newport, 1845) (specimens used by Koch and Edgecombe 2008). The alignment of the bulges is further described and proposed as an additional character state (character 4).

The examination of additional taxa within Lithobiidae revealed more variation in the shape of the median spine field than previously described and having surveyed more species we include additional character states to those already described by Koch and Edgecombe (2008) (character 5).

Although differences in shape of the bristles on the labral flap were briefly mentioned by Koch and Edgecombe (2008), our study unveiled four consistent states in the shape of bristles and transition of those from laterally to medially, which serves as a new multistate character for Lithobiidae (character 6). A transition of bristles from plumose to ‘fan-shaped’ was described for *Pleurolithobius patriarchalis* (Berlese, 1894) (Koch and Edgecombe 2008), as in the majority of the investigated species in the present study. In contrast, only ‘fan-shaped’ bristles are observed in the lithobiid *Harpolithobius anodus* (Latzel, 1880) and the henicopid Lamyctes (Lamyctes) emarginatus (Newport, 1844). On the other hand, the interpretation that Lithobius (Monotarsobius) holstii (Pocock, 1895) possesses only ‘fan-shaped’ bristles (Fig. 6E in Koch and Edgecombe 2008) seems erroneous as their figure reveals a pattern in accordance with the other examined *Monotarsobius*-species, which exhibit a transition from plumose to ‘fan-shaped’ bristles (e.g. *L. aeruginosus*, Fig. 9C).

Generally, the median sensilla cluster borders or overlaps marginally with the lateral field of spines in Lithobiomorpha (Koch and Edgecombe 2008). However, we observed a median sensilla cluster considerably expanding along the length of the lateral spine fields on the epipharynx in *E. grossipes* for Lithobiomorpha (Fig. 10D). This was also verified in *E. fasciatus* (specimens used by Koch and Edgecombe 2008), which also displays a large but partial overlap.

As mentioned in the introduction, the hypopharynx as a short outgrowth with a median crest is an apomorphic character for Lithobiomorpha. This is verified in all examined lithobiid species. Moreover, the median crest margin of all studied species of the subgenus Monotarsobius displays flattened spines (character 8) as previously described for *L. holstii* (Koch and Edgecombe 2008).

### Variability of the peristomatic structures in Lithobiidae

Besides the well-defined characters listed in the previous paragraph, our investigation also yielded several structures with high variability in appearance and/or intermediate forms between and even within species. For example, the branching bristles of (i) the labral bristle band on the distal bar, (ii) the spines of the median spine field of the epipharynx and (iii) the branching bristles as a tuft on the hypopharynx occur with several non-definable forms. Koch and Edgecombe (2008) described a smooth transverse bulge for Lithobiidae, which we confirmed for most of the examined species. However, we also observed a longitudinal striation of the whole bulge surface or at least on the lateral parts of the bulge for some species (Figs 3C, 4D). A similar description of the latter state was observed for the henicopid *Lamyctes emarginatus*, where more defined longitudinal grooves occur (Koch and Edgecombe 2008).

The paired oblique rows of elongated lateral spines on the clypeal part of the epipharynx were also considered as an apomorphic character for Lithobiidae (Koch and Edgecombe 2008). This is also confirmed in all examined lithobiid species we studied. However, the proposed character states, i.e. (2): oblique rows of single spines and (3): a few small groups of branching spines for the lateral field of spines on Lithobiidae were not consistent across the species we examined and showed many intermediate states. On this basis we excluded the character for conclusions on the systematics in Lithobiidae, especially *Lithobius*, in our study.

Koch and Edgecombe (2008) recorded groups of lateral fields of spines in the subgenus Monotarsobius in contrast to pairs of oblique rows in the rest of Lithobiidae (character 32, state (3)). These spines seem to be arranged in oblique rows as in the rest of Lithobiidae in the species L. (Monotarsobius) aeruginosus and L. (Monotarsobius) curtipes (Fig. 10F–G).

A correlation between the number of ‘bottle-shaped’ epidermal glandular shafts of Lithobiomorpha and body size was also mentioned by Koch and Edgecombe (2008), implying that larger species tend to have higher numbers. Here, we suggest the same for the number of glandular shafts, sensilla in the median sensilla cluster and the ‘nipple-shaped’ sensilla cluster, lateral spines, and the hypopharyngeal spines. This size correlation needs to be confirmed by morphometrics and statistical analysis but the phylogenetic significance of these characters is cast into doubt.

### Assumptions on the relationship of *Disphaerobius* with (sub)genera *Lithobius* and *Ezembius*

The peristomatic structures of *H. plumatus* and L. (Ezembius) giganteus described by Koch and Edgecombe (2008) and *D. loricatus* examined in this study, i.e. a missing transverse bulge (character 3), simple bristles on the labral flap of the epipharynx (character 6) and scales on the distal tips of the lips of the hypopharynx (Fig. 17E), differ from all other studied species of *Lithobius*, including L. (Ezembius) electus, even if the latter is correctly placed in the subgenus Ezembius. Several taxa in Central Asia, also species of the *giganteus*-group of *Lithobius* (Eason 1983, 1986) and of the genus *Hessebius* Verhoeff, 1941 share some morphological characters with the genus *Disphaerobius* Attems, 1926, as mentioned by Farzalieva et al. (2017): “… functionally biarticulated tarsi of leg 1–13, the antennae composed of 20 antennomeres, the rounded posterior angles of all tergites, the 1-segmented male gonopods, and Tömösváry’s organ being equal in size to the nearest ocellus or smaller.” In contrast to the three other species of the *giganteus*-group of *Lithobius*, L. (Ezembius) giganteus displays secondary sexual modifications of the tergites in males similar to *Disphaerobius* (Farzalieva et al. 2017). Here, we assume that the epipharyngeal and hypopharyngeal structures may confirm a closer relationship of L. (Ezembius) giganteus to *D. loricatus* than to L. (Ezembius) electus. This relationship is inconsistent with the classification of *Disphaerobius* as a separate subfamily, Pterygoterginae Verhoeff, 1933, because that classification would render Lithobiinae, as well as *Lithobius* and L. (Ezembius) as paraphyletic groups.

## Appendix 1

**Table:** Data matrix of 8 peristomatic characters of Lithobiidae, numbered as in the text.

Species	Characters
	12345678
L. (L.) agilis	111(0?)1000
L. (L.) calcaratus	11212000
L. (L.) carinatus	10111000
L. (L.) castaneus	10102000
L. (L.) cyrtopus	01110101
L. (L.) dentatus	11112000
L. (L.) erythrocephalus	11112001
L. (L.) fagei	10101000
L. (L.) forficatus	10100001
L. (L.) lapidicola	01112001
L. (L.) latro	11112001
L. (L.) lucifugus	11202101
L. (L.) macilentus	11111001
L. (L.) mutabilis	01112001
L. (L.) muticus	00110001
L. (L.) nodulipes	111110?1
L. (L.) peregrinus	10100200
L. (L.) piceus	10112200
L. (L.) pelidnus	11102101
L. (L.) pyrenaicus	00100001
L. (L.) tenebrosus	0121200?
L. (L.) tricuspis	101022?0
L. (L.) validus	10101001
L. (M.) aeruginosus	01112001
L. (M.) austriacus	01111001
L. (M.) crassipes	01112001
L. (M.) curtipes	10111001
L. (M.) franciscorum	(1?)1110001
L. (S.) burzenlandicus	0111?000
L. (S.) microps	01112100
L. (S.) trebinjanus	11111001
L. (E.) electus	10102001
*N. aztecus*	11100011
*D. loricatus*	100-0311
E. (E.) grossipes	10200010
